# Life Cycle Assessment of Synthesis Route and Regenerative Application of Novel GO/ZIF-60/CoNiAl-LTH Nanocomposite for Efficient Remediation of Ciprofloxacin Contaminated Water

**DOI:** 10.3390/antibiotics15060566

**Published:** 2026-06-02

**Authors:** Ishraq H. Alhamed, Aeshah AlAmri, Nuhu Dalhat Mu’azu, Malak Yahya Alkhaldi, Rawan Abdullah Bashanaini, Mohamed S. Gomaa

**Affiliations:** 1 Department of Chemistry, College of Science, Imam Abdulrahman Bin Faisal University, P.O. Box 1982, Dammam 31441, Saudi Arabia; 2230700017@iau.edu.sa (I.H.A.); 2200009016@iau.edu.sa (R.A.B.); 2 Department of Environmental Engineering, College of Engineering, Imam Abdulrahman Bin Faisal University, Dammam 31451, Saudi Arabia; 3 Department of Chemistry, King Fahd University of Petroleum and Minerals, Dhahran 31261, Saudi Arabia; malak.alkhaldi@kfupm.edu.sa; 4 Department of Pharmaceutical Chemistry, College of Pharmacy, Imam Abdulrahman Bin Faisal University, P.O. Box 1982, Dammam 31441, Saudi Arabia; msmmansour@iau.edu.sa

**Keywords:** ciprofloxacin aqueous removal, novel nanocomposites, emerging contamination remediation, adsorption mechanisms, persistent organic contaminants, environmental impact assessment, pharmaceutical-contaminated water, metal-organic framework composite (MOF)

## Abstract

**Background/Objectives**: The widespread presence of antimicrobial-resistant pharmaceutical contaminants in wastewater poses serious ecological and public health risks and remains difficult to address using conventional treatment technologies. Moreover, remediation strategies often involve overlooked environmental burdens, highlighting the need for technologies that are both efficient and environmentally sustainable. This study developed a novel GO/ZIF-60/CoNiAl -LTH (GO/ZIF-60/LTH) ternary nanocomposite adsorbent for removal of ciprofloxacin (CIP) from water matrixes while evaluating its environmental implications using Life cycle assessment (LCA). **Methods:** The adsorbent was synthesized by integrating graphene oxide (GO) and Ni–Al–Co layered triple hydroxide (LTH) into a ZIF-60 framework. Structural and surface characterization was conducted using XRD, FTIR, SEM–EDX, BET, and UV–Vis analyses. The adsorbent’s CIP aqueous uptake was evaluated through batch experiments supported by kinetic, isotherm, thermodynamic, and response surface methodology (RSM) analyses. Environmental performance was assessed through life cycle-based evaluation. **Results**: The composite achieved a maximum adsorption capacity of 291 mg g^−1^ and 91.6% removal efficiency with adsorption following pseudo-first-order kinetics and the Freundlich isotherm. The process was spontaneous and exothermic, with 75% efficiency retained after three regeneration cycles. The LCA revealed an overall global warming impact of 0.953 kg CO_2_ eq per functional unit, with the NiAlCo-LTH synthesis stage (1.04 kg CO_2_ eq) as the dominant hotspot, followed by final composite formation stage (0.66 kg CO_2_ eq). Adsorption and regeneration provided credits (−0.336 and −0.513 kg CO_2_ eq), offsetting the upstream impacts. **Conclusions:** The study demonstrates a new MOF–GO–LTH hybrid adsorbent with high CIP removal efficiency combined with its environmental sustainability assessment, providing a more comprehensive basis for adsorbent evaluation. Although the NiAlCo-LTH component was primarily responsible for the enhanced adsorption performance, yet, it also constituted the major environmental hotspot during its synthesis. These findings highlight the relevance of trade-off between functionality and environmental burden for process optimization, cleaner production, and the sustainable development of advanced adsorbents for pharmaceutical-contaminated water treatment.

## 1. Introduction

**Figure 1:**
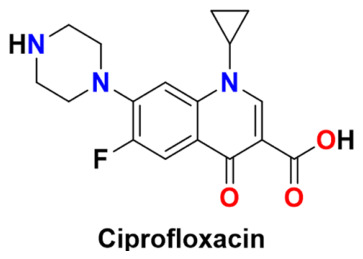
Molecular structure of ciprofloxacin.

The growing consumption of antibiotics in human healthcare and livestock production, coupled with inadequate removal during wastewater treatment, has led to their widespread occurrence in aquatic environments [1,2]. This poses significant environmental and public health concerns, primarily due to the development of antimicrobial resistance and the disruption of natural microbial communities within the ecosystem [3]. Among antibiotics, ciprofloxacin (CIP) is of a particular concern within the class of fluoroquinolone antibiotic because it is highly soluble and remains stable while exhibiting resistance to conventional wastewater treatment methods [4]. Several advanced technologies exist for removal of fluoroquinolone antibiotics from water, including membrane filtration, advanced oxidation processes, electrochemical degradation, biological treatment methods etc., [4]. These methods still face several challenges because they may produce harmful byproducts, require expensive operations and/or fail to eliminate persistent pollutants effectively [5]. Figure 1 shows the molecular structure of CIP, a widely used fluoroquinolone antibiotic characterized by a fluorinated quinolone core, a piperazine ring, and a carboxylic acid functional group. These functional groups contribute to its environmental persistence and resistance to conventional treatment processes, while also enabling interactions with adsorbents through such as electrostatic attraction, hydrogen bonding, π–π stacking, and metal–ligand complexation. This renders adsorption-based remediation method as one of the most promising approaches for its effective removal from water. Consequently, adsorption has emerged as one of the most attractive alternatives, offering high efficiency, low costs, and minimal secondary pollution [6,7]. In recent years, metal–organic frameworks (MOFs), especially zeolitic imidazolate frameworks (ZIFs), have attracted significant attention. ZIFs consist of metal ions that relate to imidazolate-based linkers. In the literature, there are several different ZIFs. The main differences between them are the used metal ions as well as the organic linkers. ZIF-8 is composed of 2-methylimidazole and Zn^2+^ ions, while ZIF-67 has a similar sodalite topology but is based on Co^2+^ ions instead of Zn^2+^. In contrast to these frameworks, ZIF-60 have completely different framework structures and different pore characteristics. The main differences are based on the used linkers as well as on the used synthesis conditions. Due to their structural characteristics, the ZIFs are more or less suitable for certain applications [8]. The most common fields of application are adsorption, catalysis, and membrane applications. The environmental application research on ZIF-8 and ZIF-67 has been extensive, but ZIF-60 with Zn(II) and imidazole linkers has been subject to limited study despite its promising structural characteristics [9]. Reported research works indicates ZIF-60 demonstrates superior adsorption capabilities because of its large surface area and its accessible microporous structure [10]. The use of pristine ZIFs in water-based applications faces challenges because they do not disperse well and show weak attraction to target pollutants. The incorporation of graphene oxide (GO) into MOF structures creates GO/MOF composites, which demonstrate better adsorption properties because of π–π stacking, hydrogen bonding and surface functionalization enhancement [11]. The composite becomes more hydrophilic because of GO addition, while the oxygen functional groups enhance pollutant adsorption capabilities [12]. The adsorptive performance of the material can be improved by adding layered triple hydroxides (LTHs), which contain Ni, Al, and Co metals. High anion-exchange capacity, adjustable interlayer spacing, and strong electrostatic interactions in the materials make them suitable for removing charged pollutants, including antibiotics [11].

Based on the forgone, this study aims to synthesize and compare three adsorbent systems, ZIF-60, GO/ZIF-60, and GO/ZIF-60/LTH, for the removal of CIP from water. The combination of Ni–Al–Co metals within layered triple hydroxide (LTH) shows promise as an additive synergetic nanocomposite material. The materials possess three key features that enhance their ability to capture negatively charged and polar substances through electrostatic attraction and hydroxyl-rich surfaces and high anion-exchange capacity [13]. The combination of LTHs with ZIFs and GO in hybrid composites remains underexplored despite their strong surface activity, which makes them an important area for future research. In the current literature surveyed, there is no reported work that explores the synthesis of a single ternary composite that combines ZIF-60 with GO and NiAlCo-LTH and its applications as a ternary composite for CIP removal from water. The new material system demonstrates promising results by integrating four different adsorption mechanisms: hydrogen bonding, π–π stacking, electrostatic forces, and metal center coordination [14,15].

On the other hand, despite the excellent adsorption performance reported for many nanocomposite adsorbents, the environmental sustainability of their synthesis routes and environmental remediation application remains a critical concern. The production of hybrid nanomaterials often involves energy-intensive processes, extensive chemical treatments, and the use of metal precursors, which can contribute significantly to environmental burdens [16]. Therefore, it is essential to evaluate whether improvements in wastewater treatment efficiency are achieved without compromising environmental integrity. Life Cycle Assessment (LCA) has emerged as a robust and standardized tool for quantifying environmental impacts across the entire life cycle of materials, including production, processing, and end-of-life stages [16,17,18]. In recent years, LCA has been increasingly applied to adsorbents, bio-based materials, and nanocomposites to identify environmental hotspots and guide the development of more sustainable synthesis strategies [16,17,19]. However, until date, studies that integrate adsorption performance with life cycle-based environmental evaluation for advanced hybrid nanocomposites remain still limited in literature.

This study had four primary objectives: (1) to synthesize and characterize pristine ZIF-60 and its binary and ternary composites with GO and NiAlCo-LTH; (2) to evaluate their adsorption performance for CIP removal under varying operating conditions; (3) to optimize the adsorption process using Response Surface Methodology (RSM); and (4) to investigate the adsorption kinetics, equilibrium isotherms, and thermodynamic behavior. In addition, a cradle-to-gate LCA was conducted to quantify the environmental impacts associated with material synthesis, application, and regeneration, enabling the identification of key environmental hotspots and required trade-offs for achieving sustainability. Thus, the novelty of this work lies not simply in the development of a GO–MOF–LTH hybrid material, but as the first reported integration of graphene oxide (GO) and Ni–Al–Co layered triple hydroxide (LTH) within a ZIF-60 framework for CIP removal from water. This multifunctional ternary composite combines the adsorption advantages of GO, the porous structure of ZIF-60, and the abundant active sites provided by LTH, resulting in enhanced adsorption performance through multiple synergistic mechanisms. Furthermore, unlike most previous studies that focus solely on adsorption efficiency, the present work integrates material synthesis, adsorption optimization, mechanistic evaluation, regeneration assessment, and life cycle sustainability analysis within a single framework. By linking adsorption performance with environmental impacts, this study provides a more comprehensive and decision-relevant basis for the sustainable design, optimization, and future scale-up of advanced MOF–GO–LTH adsorbents for antibiotic wastewater remediation.

## 2. Results and Discussions

### 2.1. Characterization of Synthesized Nanocomposites

#### 2.1.1. XRD Patterns

**Figure 2:**
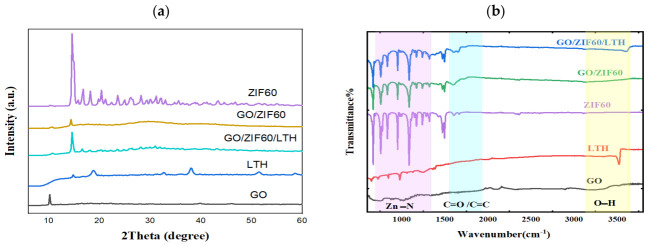
(**a**) XRD spectra and (**b**) FTIR spectra of GO, LTH, ZIF-60, GO/ZIF-60, GO/ZIF-60/LTH.

The X-ray diffraction (XRD) analysis in Figure 2a demonstrates the crystallographic arrangement of single components and the resulting composite materials. The XRD pattern of GO displays a broad diffraction peak at 2θ = 11.2°, which proves the existence of oxygen-based functional groups and disordered graphitic layers in oxidized graphene sheets [20]. The XRD pattern of LTH shows multiple weak and broad diffraction peaks, which confirm its semi-crystalline or poorly ordered phase. The XRD pattern reveals that LTH material exists as an amorphous or partially crystalline substance because it originates from hydroxyl-rich regions and contains diverse components [21]. The ZIF-60 sample displays six intense diffraction peaks at 2θ positions of 7.3°, 10.4°, 12.6°, 14.8°, 16.5°, and 18.0°, which confirm the sodalite-type crystalline structure of ZIFs. The high peak intensity together with well-defined peak shapes indicates that researchers successfully produced a highly crystalline ZIF-60 phase during their synthesis process [22]. The XRD pattern of the GO/ZIF-60 hybrid displays ZIF-60 peaks with reduced intensity and wider peak widths, which proves the effective incorporation of GO into the ZIF matrix. The interactions between GO sheets and ZIF nanocrystals during synthesis might have affected crystal growth, which resulted in reduced crystallinity. The XRD pattern of GO/ZIF-60/LTH shows ZIF peak broadening and intensity decreases, mainly in the low-angle region following LTH functionalization.

The incorporation of LTH material brings amorphous characteristics to the composite, which disrupts the ordered arrangement of the ZIF framework. The core crystalline structure of ZIF-60 remains detectable through its reflections at 7.3°, 10.4°, and 12.6° within the ternary composite structure. The GO/ZIF-60 and GO/ZIF-60/LTH composites demonstrate successful synthesis because their individual components preserve their original structures while forming a single hybrid network. The ZIF-60 crystallinity in the composite remains intact because LTH and GO amorphous features generate surface heterogeneity, which could boost adsorption capabilities.

#### 2.1.2. FTIR

FTIR spectroscopy results in Figure 2b show the functional groups of individual materials and their composites. The FTIR spectrum of GO shows a wide absorption peak at ~3430 cm^−1^ that proves the existence of hydroxyl groups on its surface. The FTIR spectrum shows two distinct peaks at ~1720 cm^−1^ and ~1620 cm^−1^, which match the C=O stretching of carboxyl and carbonyl groups and C=C skeletal vibrations of GO’s oxidized carbon structure [23,24,25]. The LTH material shows two main absorption peaks at 3410 cm^−1^ for O–H stretching and two weak peaks at ~1630 cm^−1^ that result from adsorbed water [24]. The spectral region shows no strong peaks because this material remains mostly inactive. The FTIR spectrum of pristine ZIF-60 shows distinct peaks between 1580 and 1580–1400 cm^−1^ which represent imidazolate ring vibrations, and bands between 1140 and 900 cm^−1^ that indicate Zn–N stretching modes from the ZIF framework. The observed spectral peaks confirm that ZIF structure formation was successful [23].

The GO/ZIF-60 spectrum shows ZIF-60 peaks, but these peaks shift and broaden between 1000 and 1700 cm^−1^ due to physical or weak chemical bonding between GO and the ZIF matrix [24]. The ZIF structure appears to reduce the C–O and O–H bands in GO through partial reduction and surface coverage [20,23]. The GO/ZIF-60/LTH composite shows all functional groups present in its individual components. The spectrum shows a wide band at 3415 cm^−1^, which results from the combination of O–H and N–H stretching vibrations. The imidazole structure of ZIF-60 remains intact based on the 1550–1400 cm^−1^ peaks, while C–O and Zn–N bonds produce bands at 1050–1100 cm^−1^. The new peaks and peak shifts between the pure components demonstrate that GO and ZIF-60 and LTH successfully form a ternary composite [21,26]. The spectral data confirm hybridization success and show evidence of hydroxyl, carboxyl, imidazole, and Zn–N functional groups, which can enhance organic pollutant adsorption through electrostatic forces and hydrogen bonding and π–π stacking interactions.

#### 2.1.3. Morphological and Elemental Analysis (SEM and EDX)

**Figure 3:**
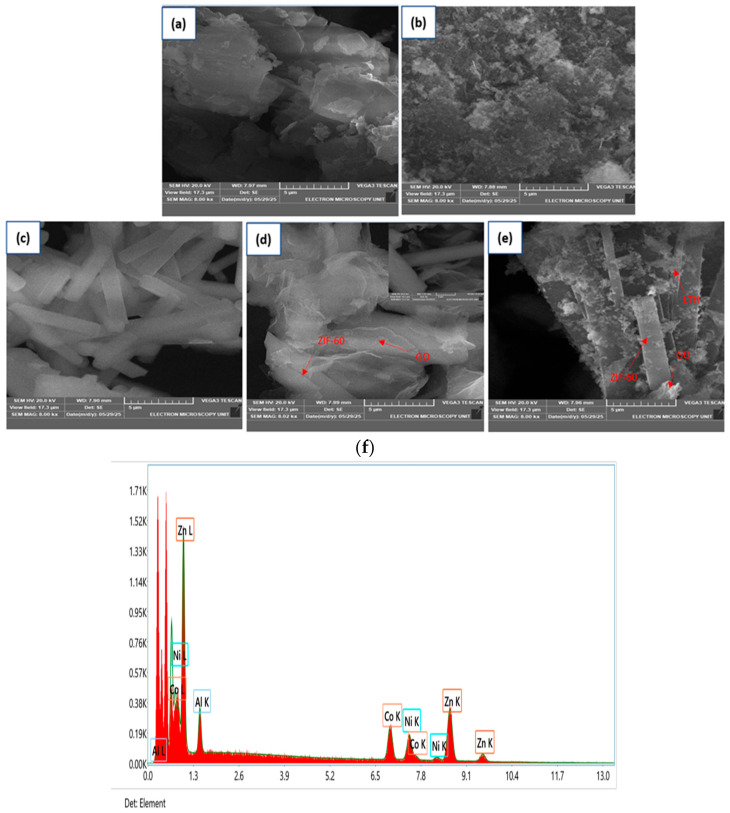
SEM images of (**a**) GO, (**b**) LTH, (**c**) pristine ZIF-60, (**d**) GO/ZIF-60 composite, and (**e**) GO/ZIF-60/LTH composite, (**f**) EDX graph of GO/ZIF-60/LTH.

The surface morphologies of synthesized materials underwent analysis through Field Emission Scanning Electron Microscopy (JEOL, Japan), which produced results shown in Figure 3a–e. The GO sample (Figure 3a) displayed its characteristic wrinkled and layered structure, which is typical for exfoliated graphene oxide sheets. The folded structure of this material creates extensive contact points, which enable effective material-to-material and material-to-pollutant interactions [23]. The LTH image (Figure 3b) shows an irregular surface with amorphous features and non-uniform agglomerates that create a heterogeneous texture. The irregular surface structure indicates the presence of hydroxyl-rich regions, surface defects, and natural cavities, which create better contact points for composite material applications [21,26]. The XRD analysis confirmed the high crystallinity of ZIF-60, which matches the rod-like and elongated crystal structure observed in Figure 3c. The ordered structures in this material indicate successful ZIF-60 framework formation and its specific structural arrangement [23].

The inset in Figure 3d highlights the embedded rod-like ZIF-60 crystals within the GO matrix, some ZIF-60 particles penetrate the GO layers. The ZIF crystals maintain their position inside GO layers because of strong bonding between the two materials. The hybrid structure enables better distribution of ZIF particles while strengthening the overall structure of the composite material [20,23]. The GO/ZIF-60/LTH ternary composite shows a dense structure in Figure 3e because LTH particles form irregular blocks that integrate into the GO/ZIF-60 network. The composite structure demonstrates successful integration of all components through its porous hierarchical structure, which enables better adsorption because of enhanced diffusion routes and better interfacial relationships [21,26]. The GO/ZIF-60/LTH sample underwent Energy Dispersive X-ray Spectroscopy (EDX) analysis to determine its elemental makeup (Figure 3f). The EDX spectrum shows that the material contains Zn from ZIF-60 together with C and O from GO and LTH and additional elements including N, Co, and Al. The presence of Zn peaks in the spectrum proves that ZIF structures exist in the composite, while C, O, and N signals indicate functional groups that can bind to pollutants. The minor peaks of Al and Co stem from LTH components and unclean precursors, which prove the intricate nature of the ternary hybrid material. The successful creation of a multicomponent composite with integrated porous structures and active surfaces is confirmed by these findings, which make GO/ZIF-60/LTH suitable for wastewater treatment operations.

#### 2.1.4. BET Surface Area and Porosity Analysis

The textural properties of the synthesized materials were evaluated using nitrogen adsorption–desorption isotherms at 77 K, and the results are summarized in Table 1. The pristine ZIF-60 exhibited a remarkably high BET surface area of 4213.4 m^2^/g, consistent with its highly porous and crystalline structure. Its pore volume and average pore diameter were measured at 4.059 cc/g and 1.92 nm, respectively, indicating a predominantly microporous framework typical of zeolitic imidazolate materials [27]. Upon incorporation of graphene oxide (GO), the resulting GO/ZIF-60 composite showed a substantial reduction in surface area to 646.6 m^2^/g and a pore volume of 0.478 cc/g, with a decreased average pore size of 0.553 nm. This decline may be attributed to partial blockage of ZIF-60 micropores by GO sheets and the possible collapse of some porous domains during synthesis. Nevertheless, the enhancement of surface functional groups from GO may have introduced favorable adsorption sites for CIP.

Further incorporation of layered double hydroxides (LDH, specifically NiAlCo-LTH) to form GO/ZIF-60/LTH yielded a material with a BET surface area of 568.9 m^2^/g, a pore volume of 0.281 cc/g, and a notably increased average pore diameter of 0.987 nm. Although the surface area decreased slightly compared to GO/ZIF-60, the enlarged pore size indicates the emergence of mesoporosity, which is advantageous for the diffusion and interaction of larger antibiotic molecules such as CIP. This adjustment to the structure may help explain why the GO/ZIF-60/LTH hybrid worked better in later adsorption tests [28]. These findings confirm that the addition of GO and LTH alters the textural characteristics of ZIF-60, with trade-offs between surface area and pore accessibility. The BET results provide strong evidence that hierarchical porosity and surface chemistry synergistically influence the adsorptive capabilities of the hybrid materials.

#### 2.1.5. UV–Vis Spectroscopic

**Figure 4:**
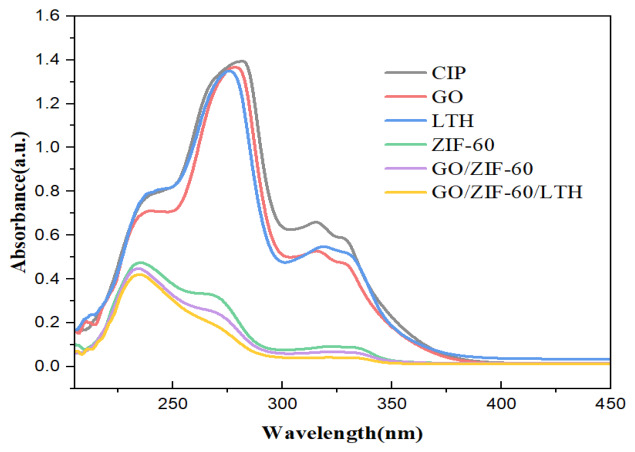
UV-Vis absorption spectra of pure CIP, GO, ZIF-60, LTH, GO/ZIF60, and GO/ZIF60/CoNiAl-LTH nanocomposites (Concentration = 30 ppm, initial pH = 6, temperature = 25 °C, Time = 24 h).

The UV–Vis spectroscopic analysis in Figure 4 shows how CIP interacts electronically with GO and LTH and ZIF 60 and GO/ZIF 60 and GO/ZIF 60/CoNiAl-LTH composite materials. The two peaks at ~275 nm and ~320 nm in the pristine CIP solution correspond to its aromatic and carboxyl chromophores. The adsorption process leads to significant peak intensity reduction in all tested materials which proves their ability to remove CIP from solution. The GO/ZIF 60/CoNiAl-LTH composite shows the most effective absorbance reduction at ~275 nm which indicates it has the best removal performance among all tested materials. The strong absorbance reduction indicates strong adsorption behavior because the material combines π–π stacking between GO and CIP aromatic rings with hydrogen bonding from LTH hydroxyl and carboxyl groups and metal–ligand coordination from ZIF 60 active sites [29]. The lack of λ_max shift in the spectra indicates that physical adsorption dominates over chemical modification of c CIP structure during the interaction process. The observed spectral changes between the drug and composite materials prove that GO/ZIF 60/CoNiAl-LTH functions as an efficient adsorbent for pharmaceutical removal from water solutions.

#### 2.1.6. Effect of pH on CIP Adsorption

**Figure 5:**
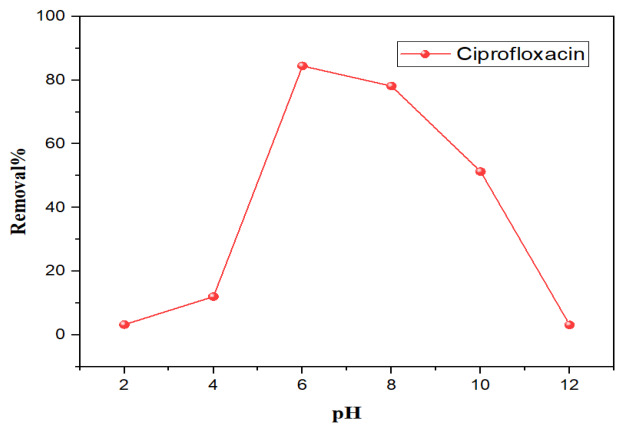
Effect of pH on the removal efficiency of ciprofloxacin using the GO/ZIF60/CoNiAl-LTH.

The adsorption performance of adsorbent materials depends heavily on solution pH because it affects both the ionization state of adsorbates and the surface charge of adsorbents. In this study, we investigated CIP removal efficiency at different pH levels to find the optimal condition that produced the highest adsorption results. The experimental findings showed that adsorption behavior strongly depends on pH levels because the highest removal rates occurred at pH 6 (Figure 5).

The adsorption behavior of CIP occurs best at pH 6 because the zwitterionic form of the compound emerges at this pH value. The zwitterionic form CIP at pH 6 enables strong electrostatic and hydrogen bond interactions with adsorbent surface functional groups. The surface charge of GO/ZIF-60/LTH at this pH range is either positive or neutral, which enables better attraction to the negatively charged parts of zwitterionic ciprofloxacin molecules. The adsorption process becomes less effective when the adsorbent surface becomes too protonated at pH values below 5 because of electrostatic repulsion and when functional groups deprotonate above pH 7, which reduces electrostatic attraction between the adsorbent and the antibiotic [30,31,32]. The results match previous studies about MOF and GO adsorbents, which demonstrate that pH control enables the achievement of optimal adsorption outcomes. The observed behavior demonstrates that both the adsorbent surface chemistry and antibiotic speciation need evaluation for achieving optimal removal efficiency in real-world environmental applications.

### 2.2. RSM Modeling of Ciprofloxacin Removal from Water

#### 2.2.1. RSM Development and Optimization

The GO/ZIF60/CoNiAl-LTH was chosen for further optimization through RSM modeling because it demonstrated the highest CIP removal capacity and operational stability across different conditions during preliminary batch adsorption tests among all synthesized composites. The material achieved its enhanced performance through its synergistic structure, which united ZIF-60′s high surface area and porosity with GO functionalization and LTH post-synthetic thermal activation. As per Table 2, Box–Behnken Design (BBD) experimental design was adopted for implementing the RSM modeling and optimizing CIP removal from which seventeen (17) experimental required runs [33] were undertaken to study influence of contact time on removal efficiency while keeping adsorbent dose and pH constant of 6. The RSM modeling, statistical analysis and optimization was undertaken with the aid of statistical package, Design Expert 8 software [33]. The regression developed model given in Equation (1) is expressed in terms of coded variables generated by the Box–Behnken design, where A, B, and C represent contact time, initial CIP concentration, and temperature, respectively. The coded levels of −1, 0, and +1 correspond to the low, center, and high experimental data point levels: contact time (60, 180, and 300 min), concentration (10, 30, and 50 ppm), and temperature (25, 35, and 45 °C), respectively. The model achieved high predictability indicated by its *R*^2^ = 0.974 and adjusted *R*^2^ = 0.941. The predicted *R*^2^ of 0.8623 is in reasonable agreement with the adjusted *R*^2^, i.e., the difference is less than 0.2 while its adequate precision ratio of 14.10, which exceeded the required threshold of 4 [33]. Accordingly, the model shows high reliable performance for predicting the experimental data representation because of its consistent high *R*^2^ value, adjusted *R*^2^ value, and predicted *R*^2^ value, indicating the model’s suitability as further confirmed by the lack-of-fit non-significant *p*-value of 0.0082 [34].

(1) $$\mathrm{Removal Efficiency}\;(\%)\;=+89.83\;+\;0.1086\;A\;-\;5.47\;B\;+\;0.6483\;C\;-\;0.1875\;\mathrm{AB}\;+\;0.0349\;\mathrm{AC}\;-\;0.0625\mathrm{BC}\;-\;4.88\;A^{2}\;+\;\;0.8842\;B^{2}\;-\;3.77\;C^{2}$$

where A, B, and C represent the coded values for contact time, concentration, and temperature, respectively.

**Figure 6:**
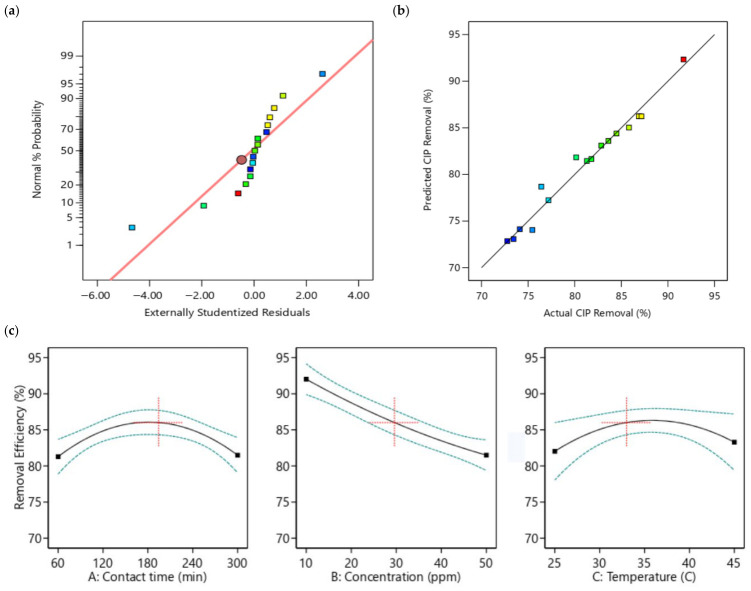
(**a**) Normal probability plot of residuals for CIP removal; (**b**) plot of the relationship between the theoretical and experimental values of CIP removal (**c**) Impacts of individual factors (A: contact Time; B: concentration; C: Temperature) on CIP removal.

**Figure 7:**
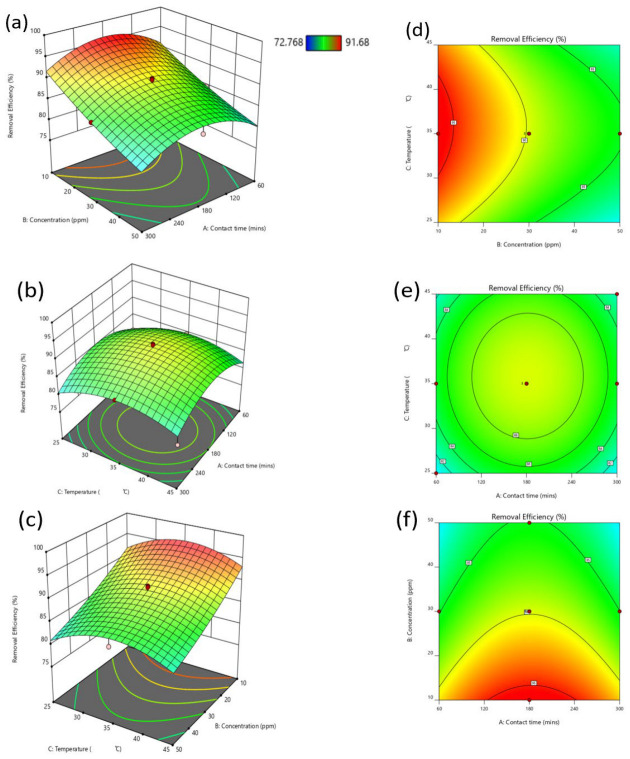
Three-dimensional response surface plots (**a**–**c**) and corresponding 2D contour plots (**d**–**f**) representing the interactive effects of contact time, Ciprofloxacin concentration, and temperature on the removal efficiency by GO/ZIF60/CoNiAl-LTH composite.

The model residuals in Figure 6a show a random distribution with symmetry around zero, which indicates no major systematic errors or biases in the model. The experimental removal values match well with the predicted values, which appear as a tight cluster around the diagonal line in Figure 6b. The model demonstrates strong predictive capability because its statistical approach produces accurate results that match experimental data. The model demonstrates reliable predictive capabilities for ciprofloxacin removal optimization under different operational settings because its predicted values match experimental results precisely [34]. The 2D contour plots (Figure 7d–f) demonstrate how different variables affect each other. The combination of longer contact periods with lower pollutant concentrations leads to better removal efficiency according to the contact time and concentration interaction results. The contact time vs. temperature plot shows that adsorption capacity reaches its peak when temperatures range between 30 and 35 °C and contact time extends. The results indicate that optimal removal occurs when the concentration levels are low and the temperature falls within an intermediate range. The optimization analysis determined that the best operating conditions would be 240 min of contact time with 10 ppm ciprofloxacin at 30 °C to reach a predicted removal efficiency of around 91.6%. Both the 2D contour and 3D response surface plots demonstrate how operational variables affect ciprofloxacin removal efficiency through their combined influence. The removal efficiency of the adsorption process increased when contact time was extended and initial concentration levels decreased (Figure 7a). The adsorption process improved when the interaction time was increased because it enables equilibrium establishment and maximizes the utilized available active sites for adsorption.

Meanwhile, the contact time vs. temperature plot (Figure 7c) demonstrates that removal efficiency reaches its maximum at temperatures between 30 and 35 °C because increased temperature enhances molecular movement, which leads to improved adsorbate-surface interactions between the GO/ZIF-60/LTH composite. The removal efficiency decreased at high temperatures because of desorption effects. The enhanced removal efficiency observed at moderate temperatures (30–35 °C) can be attributed to improved molecular mobility and diffusion of ciprofloxacin molecules toward the active adsorption sites. However, further temperature increases may weaken the intermolecular forces responsible for adsorption, including hydrogen bonding, electrostatic interactions, π–π stacking, and van der Waals forces. In addition, temperature-induced changes in the orientation and accessibility of GO and hydroxyl-containing functional groups may reduce the stability of adsorbent–adsorbate complexes, thereby promoting desorption and lowering the adsorption efficiency [35,36,37].

The concentration–temperature interaction shown in Figure 7c indicates that the highest removal efficiencies were achieved at low ciprofloxacin concentrations and moderate temperatures. This behavior can be attributed not only to favorable adsorption kinetics and mass transfer but also to the availability of a larger proportion of unoccupied adsorption sites. At higher pollutant concentrations, competition for active sites increases and partial saturation of the adsorbent surface occurs, reducing the overall removal efficiency despite the beneficial effect of moderate temperatures. Therefore, the observed response reflects the combined influence of temperature-dependent adsorption behavior and adsorbent site saturation. The 3D surface plots confirmed the predictive power of the model while showing the specific operating ranges that produce the highest removal rates according to the ANOVA and numerical optimization results. The RSM optimization study produced results that showed temperature affects ciprofloxacin removal through the GO/ZIF-60/LTH composite material between 25 and 45 °C. The removal efficiency showed a parabolic relationship with temperature according to the results presented in Figure 7c. The ANOVA results showed temperature was not statistically significant at 91.64% confidence, yet the response pattern demonstrated a clear trend. The removal efficiency increased when temperature rose from 25 °C to 35 °C before achieving its peak at the center point and then decreased at higher temperatures.

The adsorption process shows endothermic behavior until a specific point where CIP molecules become more mobile and penetrate deeper into the adsorbent pores for better adsorption. The adsorption process shows decreased performance because the adsorbent surface was susceptible to losing its binding strength to the adsorbate when temperatures exceed this threshold. Accordingly, the observed decrease in removal efficiency at 45 °C temperature cannot be explained by surface interactions alone. This is because, related reported research works demonstrated that CIP experiences partial thermal decomposition at elevated temperatures above 40 °C [38]. Consequently, the highest tested temperature in this study was expected to have resulted in decreased adsorption efficiency because the antibiotic molecule became structurally unstable and broke down. The degradation process creates substances that have reduced binding properties to the adsorbent material, thus reducing the removal efficiency [38]. The results match previous research on MOF-based adsorbents that demonstrated that temperature variations control the equilibrium between adsorption and desorption while also affecting the stability of temperature-sensitive adsorbates [35,36,37,39].

Thus, from the forgone, inferably, the decrease in removal efficiency observed at 45 °C is primarily attributed to reduced adsorption affinity and the weakening of adsorbent–adsorbate interactions at elevated temperatures. Higher temperatures can weaken hydrogen bonding, electrostatic interactions, van der Waals forces, and π–π interactions, thereby promoting desorption and reducing adsorption capacity [35,36,37,39]. Although thermal degradation of ciprofloxacin has been reported under certain conditions, degradation products were not monitored in this study; therefore, any contribution of degradation to the observed concentration reduction cannot be confirmed, and as such considered as a potential contributing factor [38].

While the RSM provided an effective approach for process optimization and prediction of CIP removal under controlled conditions with minimal experimental and resources requirements [21], the experiments were conducted using synthetic aqueous solutions. Therefore, further studies involving real wastewater matrices are recommended to evaluate the influence of competing ions, natural organic matter, and other co-existing contaminants on the adsorption performance and applicability of the GO/ZIF-60/LTH composite under practical treatment conditions [24].

#### 2.2.2. Kinetics of CIP Adsorption

**Figure 8:**
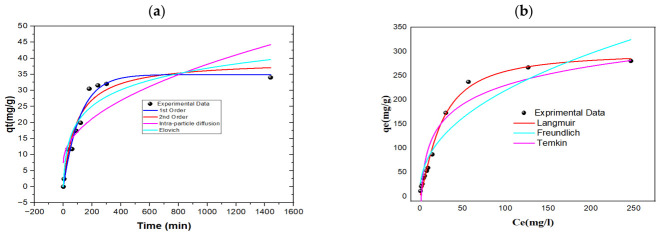
Fitting of adsorption (**a**) kinetics models and (**b**) isotherm models for experimental data for CIP adsorption onto GO/ZIF-60/LTH (Co = 30 ppm, initial pH = 7, temperature = 298 K).

To gain deeper insight into the adsorption mechanism of CIP onto the GO/ZIF-60/LTH composite, various kinetic models were applied to the experimental data, including pseudo-first-order, pseudo-second-order, intra-particle diffusion, and the Elovich model. Figure 8a shows the corresponding kinetic curves, and Table 3 [40] gives a summary of their parameters. The best fit was obtained using the pseudo-first-order model, which displayed the highest correlation coefficient (*R*^2^ = 0.97212), compared to the pseudo-second-order model (*R*^2^ = 0.9509). This strong linearity suggests that the rate-limiting step is likely governed by physisorption, where the interaction between CIP molecules and the surface-active sites occurs via weak van der Waals forces or hydrogen bonding. The calculated equilibrium adsorption capacity (q_ecal_ = 34.9 mg/g) also aligns reasonably well with the experimental values, supporting the accuracy of the model. Although the pseudo-second-order model also demonstrated a high correlation, its lower *R*^2^ value and deviation in predicted qe (39.42 mg/g) suggest it is less appropriate than the first-order model. Additionally, the Elovich model, often associated with heterogeneous surfaces, showed moderate fitting (*R*^2^ = 0.898), while the intra-particle diffusion plot did not pass through the origin, indicating that intraparticle diffusion may be involved but is not the sole rate-controlling step.

These findings are consistent with the RSM results, which revealed that contact time had a statistically significant influence on removal efficiency (*p* < 0.05). Both models and the surface plot showed that increasing time initially enhanced adsorption, but beyond a certain point, the system reached equilibrium. This synergy between RSM and kinetic modeling provides strong evidence that the adsorption process is time-dependent, with a fast initial uptake followed by a gradual approach to equilibriumThese results confirm that adsorption of CIP onto GO/ZIF-60/LTH follows a multi-step mechanism, involving rapid surface interaction followed by slower diffusion into interior pores. The kinetic profile, supported by high R^2^ values and agreement with RSM optimization, further validates the effectiveness of the composite for antibiotic removal under the tested conditions.

#### 2.2.3. CIP Adsorption Isotherm

The adsorption behavior of ciprofloxacin (CIP) onto the GO/ZIF-60/LTH composite was evaluated using three common isotherm models: Langmuir, Freundlich, and Temkin. As shown in Figure 8b and Table 4, all models exhibited reasonable fitting, but with varying degrees of correlation to the experimental data [35,39,41]. Among the three models, the Freundlich isotherm provided the best fit, as indicated by the highest correlation coefficient (*R*^2^ = 0.980). This model assumes adsorption occurs on a heterogeneous surface and supports the possibility of multilayer formation, which aligns well with the material’s structural complexity and the presence of diverse active sites introduced by the GO and LTH components. The Freundlich constants *K*_f_ (10.11 mg/g) and n (1.46) further support a favorable and efficient adsorption process (n > 1). The Langmuir model, though slightly less fitting (*R*^2^ = 0.89), still suggests a high monolayer adsorption capacity (q_max_ = 291 mg/g), indicating that uniform adsorption sites may also exist on the composite surface. This hybrid behavior combining monolayer and multilayer adsorption can be attributed to the synergistic effects of ZIF-60, GO, and LTH. The Temkin model also showed acceptable agreement (*R*^2^ = 0.9315), implying that the heat of adsorption decreases gradually with coverage due to adsorbate–adsorbent interactions. The calculated B (71.1) and KT (0.263 L/mg) reflect moderate adsorption energies, suggesting a chemisorption mechanism involving electrostatic attractions and possibly hydrogen bonding. The high q_max_ from the Langmuir model aligns with the optimized conditions predicted by RSM, under which GO/ZIF-60/LTH achieved up to 91.2% removal efficiency. This convergence between the empirical model (RSM) and the equilibrium studies (isotherms) reinforces the robustness of the adsorption system and the potential of this composite for CIP removal in real-world water treatment applications.

#### 2.2.4. CIP Adsorption Thermodynamics

**Figure 9:**
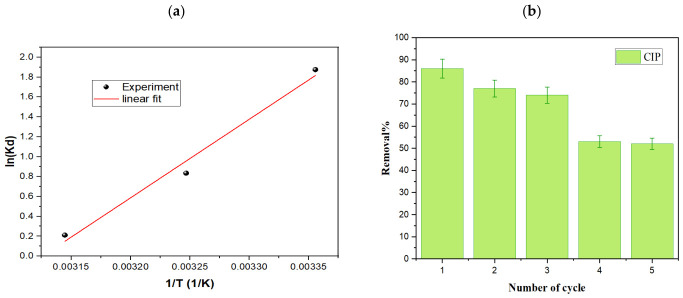
(**a**) Van’t Hoff linear plot for effect of temperature on CIP adsorption onto GO/ZIF-60/LTH (Co = 30 ppm, initial pH = 7, temperature = 298 K) and (**b**) Reusability of GO/ZIF-60/LTH adsorbent for ciprofloxacin removal over five successive adsorption cycles.

To better understand the adsorption mechanism of CIP onto the GO/ZIF-60/LTH composite, thermodynamic parameters including Gibbs free energy change (ΔG°), enthalpy change (ΔH°), and entropy change (ΔS°) were calculated based on the Van’t Hoff plot shown in Figure 9a. The linear fit of ln(Kc) versus 1/T exhibited a good correlation, allowing the estimation of ΔH° and ΔS° from the slope and intercept, respectively [36]. As given in Table 5, t all the tested temperatures the negative values of ΔG° (−4.81 to −0.56 kJ/mol), indicate that the adsorption process is spontaneous and thermodynamically favorable [37]. The magnitude of ΔG° becomes less negative with increasing temperature, indicating that spontaneity decreases as temperature rises. This trend is consistent with physical adsorption, which typically weakens at elevated temperatures.

Furthermore, the enthalpy change (ΔH°) was found to be −68.37 kJ/mol, which, while negative, indicating the adsorption of ciprofloxacin onto GO/ZIF-60/LTH increasing temperature less favors the adsorption capacity, i.e., an exothermic adsorption process [37]. The negative ΔS° (−214.16 J/mol·K) implies a decrease in randomness at the solid–liquid interface during the adsorption, possibly due to strong interactions or ordering of the CIP molecules onto the adsorbent surface [37]. As observed earlier in RSM study, the adsorption efficiency increased from 25 to approximately 30–35 °C, likely due to enhanced molecular mobility and improved mass transfer of ciprofloxacin molecules toward the active adsorption sites. However, a further increase to 45 °C resulted in a decline in removal efficiency. This behavior is consistent with the thermodynamic results, which indicate an exothermic adsorption process (ΔH = −68.37 kJ mol^−1^). At elevated temperatures, the weakening of adsorbent–adsorbate interactions, including hydrogen bonding, electrostatic attraction, van der Waals forces, and π–π interactions, may reduce adsorption affinity and promote desorption. The negative entropy change (ΔS = −214.16 J mol^−1^ K^−1^) further suggests a decrease in interfacial randomness during adsorption, supporting the formation of a more ordered adsorbent–adsorbate system. Although, CIP degradation byproducts were not monitored, it is speculated that there is probably potential contribution of ciprofloxacin degradation as observed at the elevated temperature [35,36,37,39].

#### 2.2.5. The Recyclability of Adsorbents

Figure 9b illustrates the recyclability and reusability behavior of the synthesized GO/ZIF-60/LTH nanocomposite across five consecutive adsorption cycles for ciprofloxacin (CIP) removal. Initially, the material exhibited high removal efficiency, exceeding 85% during the first cycle. However, a gradual decrease in removal percentage was observed with each subsequent cycle, reaching approximately 52% by the 5th cycle. This declining trend is primarily attributed to the partial loss of active adsorption sites or structural degradation of the composite during the regeneration process. Additionally, residual ciprofloxacin molecules or incomplete desorption may have hindered full recovery of adsorption capacity. Despite the observed reduction, the nanocomposite retained 75% of its initial performance after the 3rd cycles, demonstrating a reasonable regeneration behavior under mild condition. The small error bars reflect consistent experimental reproducibility and suggest minimal variation between replicates. Interestingly, the regenerative desorption of the CIP uptake attached to the spent GO/ZIF-60/LTH was successfully achieved using deionized water only, without using any chemical reagent, rendering the regeneration process more sustainable.

**Figure 10:**
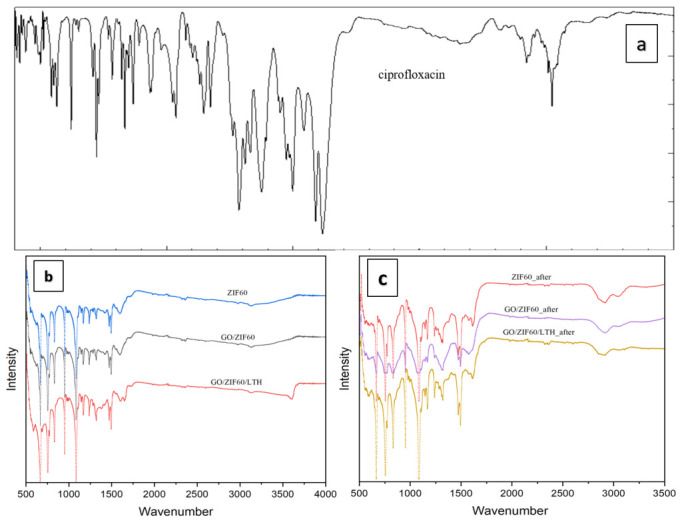
FTIR spectra of (**a**) Ciprofloxacin (**b**) ZIF60, GO/ZIF60, GO/ZIF-60/LTH before Adsorption Ciprofloxacin (**c**) ZIF60, GO/ZIF60, GO/ZIF-60/LTH after Adsorption Ciprofloxacin.

FTIR spectra analysis of the GO/ZIF-60/LTH composite before and after ciprofloxacin adsorption helps explain the adsorption mechanism of this antibiotic onto the material. The FTIR spectra show significant peak shifts and new absorption bands, which confirm the antibiotic molecules interact with functional groups on the adsorbent surface (Figure 10a–c). The FTIR spectrum of pure CIP (Figure 10a) displays distinct peaks that correspond to O–H and N–H stretching bonds and C=O groups and aromatic ring structures. The FTIR spectra of ZIF60 and GO/ZIF60 and GO/ZIF-60/LTH show three main absorption regions before adsorption: O–H stretching at ~3400 cm^−1^, C=C stretching of aromatic rings at 1600 cm^−1^, and metal–ligand coordination signals between 400 and 600 cm^−1^ [42]. The FTIR spectrum shows distinct changes after CIP adsorption (Figure 10c). The O–H stretching peak shows a small displacement because the adsorbent hydroxyl groups establish hydrogen bonds with ciprofloxacin carboxyl and ketone groups. The disappearance of C=O stretching peaks at 1720 cm^−1^ and the emergence of new bands between 1450 and 1500 cm^−1^ indicate that π–π stacking occurs between ciprofloxacin aromatic rings and the GO conjugated structure [42]. The bands associated with Zn–N or Al–O coordination shift in the spectrum indicate that CIP interacts with metal centers in the composite through chelation or metal-ligand bonding.

The spectral changes indicate that ciprofloxacin adsorption onto GO/ZIF-60/LTH occurs through multiple non-covalent forces, which include hydrogen bonding and π–π stacking and electrostatic attraction. The nanocomposite’s high surface area and hierarchical porosity enable molecular access and anchoring, while ciprofloxacin’s carboxyl, ketone, and amine groups drive the non-covalent interactions. The GO component in the material strengthens π–π stacking interactions, and LTH provides numerous active sites, which also helps maintain pH stability through adsorbent-adsorbate bonding [43,44].

#### 2.2.6. Comparative Performance Analysis

The synthesized GO/ZIF-60/LTH composite developed in this study has demonstrated excellent adsorption performance toward CIP removal from aqueous media. The material shows high adsorption capacity at 291 mg/g while removing 91.5% of antibiotics because of its powerful antibiotic-binding properties. Compared to previously reported materials (Table 6), our composite exhibits a highly competitive removal efficiency. The ZIF and LTH structure provides both increased surface area and hierarchical porosity and numerous functional groups, which enable effective CIP molecule binding. The addition of GO enables better π–π stacking and electrostatic attraction, which are crucial for antibiotic compound capture.

The results obtained confirm that the GO/ZIF-60/LTH composite not only competes with but also, in several aspects, outperforms many of the reported adsorbents in terms of both capacity and removal efficiency. The material shows potential for environmental cleanup and water purification at industrial scales, according to this assessment. While the GO/ZIF-60/LTH composite demonstrated superb adsorption performance, its practical sustainability must also consider the environmental burdens associated with material synthesis. Therefore, a LCA was conducted to evaluate whether the performance gains justify the environmental impacts of producing the hybrid adsorbent and the results presented in sections below.

### 2.3. Life Cycle Assessment for CIP GO-ZIF60-LTH

A cradle-to-gate life cycle assessment (LCA) was conducted to quantify the environmental impacts associated with the synthesis, application, and regeneration of the GO/ZIF-60/LTH system. Environmental impacts are evaluated at two complementary levels: midpoint, which reflects specific impact categories (e.g., global warming, toxicity), and endpoint, which aggregates these into broader damage categories-human health, ecosystems, and resource scarcity-providing an integrated basis for hotspot identification and process optimization [18].

#### 2.3.1. Midpoint Environmental Impact Assessments

**Figure 11:**
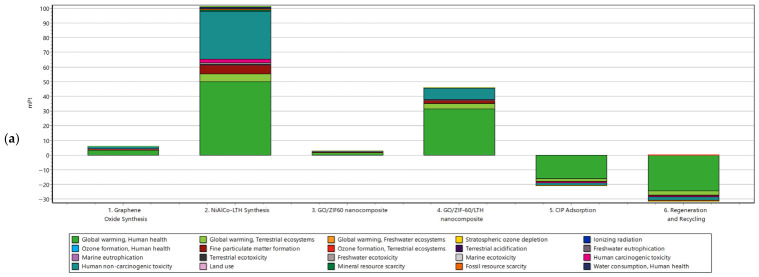

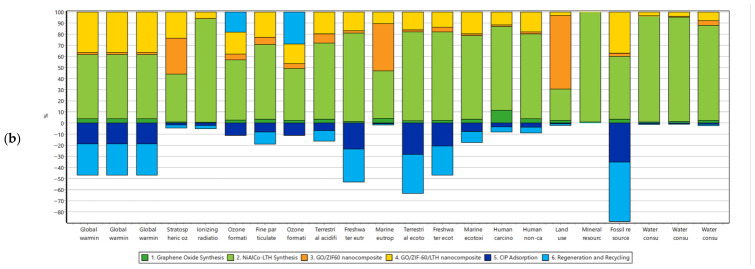
(**a**) Absolute contribution of each of the synthesis routes to the aggregated impacts (expressed in mPt) and (**b**) Relative percentage contribution of each stage across individual midpoint impact categories for the synthesis routes, adsorption, and regeneration/reusing of GO-ZIF60-LTH nanocomposite for CIP uptake from water.

The midpoint environmental impact results in Figure 11a,b reveal a clear dominance of material synthesis stages, particularly NiAlCo-LTH synthesis (Stage 2) and GO/ZIF-60/LTH composite formation (Stage 4), which together account for the majority of the environmental burden across most impact categories. As shown in Figure 11a, Stage 2 exhibits the highest overall contribution, reaching approximately 100 mPt, driven primarily by the intensive use of chemical precursors (metal salts) and associated energy requirements. This is followed by Stage 4, contributing about 45–47 mPt, equivalent to nearly 45–50% of Stage 2 impacts (Figure 10b), where additional processing, integration, and drying steps further increase energy and resource consumption.

In contrast, GO synthesis (Stage 1) and GO/ZIF-60 formation (Stage 3) contributes relatively minor impacts, with values of approximately 5–7 mPt and 2–3 mPt, respectively, representing less than 10% and 5% of the total synthesis burden. However, their contributions remain non-negligible in categories related to human toxicity, fossil resource use, and fine particulate matter formation, reflecting the influence of chemical reagents and electricity use. A notable feature of the results is the presence of negative contributions (environmental credits) associated with CIP adsorption (Stage 5) and regeneration/recycling (Stage 6), as observed in Figure 11a. Specifically, Stage 5 provides a credit of approximately −15 to −18 mPt, while Stage 6 exhibits a larger offset of about −25 to −30 mPt. These credits arise from the functional performance of the adsorbent, where pollutant removal and material reuse offset part of the environmental burdens. The regeneration stage, in particular, compensates for nearly 40–45% of the impacts associated with Stage 4, highlighting the importance of adsorbent reusability in improving overall environmental performance per functional unit.

The relative contribution analysis in Figure 11b further confirms that global warming potential, terrestrial ecotoxicity, freshwater ecotoxicity, and resource scarcity indicators are largely governed by Stage 2 (60–70% contribution) and Stage 4 (20–30% contribution). Energy consumption (especially for drying and thermal processes) and chemical usage are the primary drivers of these impacts. Meanwhile, categories such as water consumption and human toxicity show more distributed contributions across stages, although Stage 2 still dominates with approximately 50–60% contribution, followed by Stage 4 (20–30%), reflecting the influence of synthesis-related operations.

Generally, the midpoint results demonstrate that while the synthesis of advanced hybrid nanocomposites is inherently resource- and energy-intensive—particularly due to NiAlCo-LTH synthesis (100 mPt)—the adsorption efficiency and regeneration capability of GO/ZIF-60/LTH provide measurable environmental benefits, offsetting approximately 20–30% of the total lifecycle impacts. These findings highlight that process optimization efforts should focus on reducing energy consumption (especially drying) and chemical inputs during LTH and composite synthesis, while maintaining high adsorption capacity and regeneration performance.

Table 7 provides stage quantitative breakdown of midpoint environmental impacts, expressed as unweighted indicators (e.g., kg CO_2_ eq for global warming, kBq Co-60 eq for ionizing radiation, kg 1,4-DCB eq for toxicity-related categories etc), enabling identification of key hotspots. Similarly, the midpoint results clearly identify NiAlCo-LTH synthesis as the dominant environmental hotspot across nearly all impact categories. This stage alone contributes the majority of impacts, including 1.044 kg CO_2_ eq in global warming, 0.2426 kBq Co-60 eq in ionizing radiation (98% of total), and extremely high contributions to ecotoxicity indicators such as terrestrial ecotoxicity (35.469 kg 1,4-DCB eq), marine ecotoxicity (39.947 kg 1,4-DCB eq), and human non-carcinogenic toxicity (37.451 kg 1,4-DCB eq). These results confirm that metal precursor usage (Ni, Co, Al salts) and associated chemical processing are the primary environmental drivers. The second most critical contributor is GO/ZIF-60/LTH composite synthesis (Stage 4), which adds substantial impacts, particularly in global warming (0.6598 kg CO_2_ eq), marine ecotoxicity (10.547 kg 1,4-DCB eq), and human toxicity categories (8.801 kg 1,4-DCB eq). This reflects the additional burden from energy-intensive drying and composite integration steps. In contrast, GO synthesis and GO/ZIF-60 formation have comparatively minor contributions across all categories, typically accounting for less than 10% of total impacts, indicating that their influence on the overall environmental profile is limited. A critical and positive aspect of the system is the presence of significant environmental credits associated with CIP adsorption and regeneration/recycling. These stages reduce impacts across multiple categories, including −0.3356 and −0.5129 kg CO_2_ eq in global warming, and substantial reductions in ecotoxicity (e.g., −12.556 and −15.610 kg 1,4-DCB eq for terrestrial ecotoxicity). This demonstrates that adsorbent functionality and reusability offset a considerable portion of upstream burdens, particularly those associated with composite synthesis.

#### 2.3.2. Endpoint Environmental Impacts Assessments

**Figure 12:**
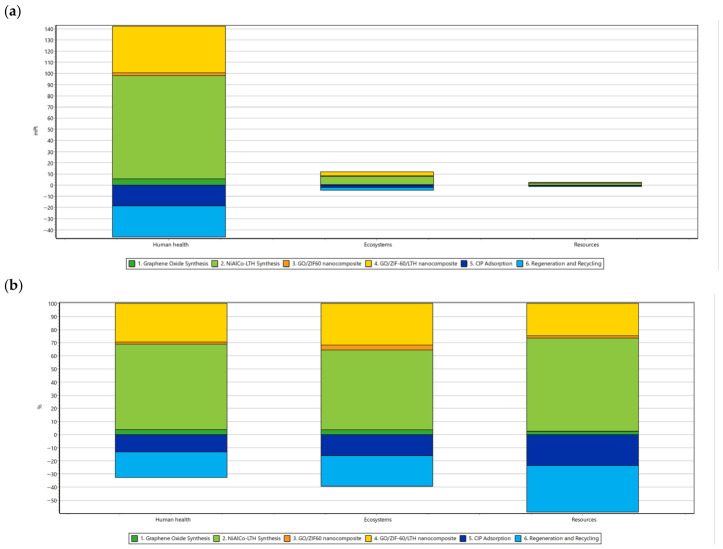
(**a**) Endpoint weighting environmental impact categories (Human Health, Ecosystems, and Resources) and (**b**) Percent relative cumulative contribution characterized impact for the synthesis and application of GO/ZIF-60/LTH for CIP uptake.

The endpoint impact assessment (Figure 12a,b) aggregated indicators into three damage categories-human health, ecosystems, and resources—providing an integrated perspective of the overall environmental burden expressed in millipoints (mPt). Similarly, as demonstrated in Figure 12a the results clearly indicate that stage 2 is the dominant contributor across all endpoint categories, followed by GO/ZIF-60/LTH composite synthesis. In the human health category, the total impact is approximately 140–145 mPt, with Stage 2 contributing the majority (90–95 mPt; 65–70%), followed by Stage 4 (35–40 mPt; 25–30%). Negative contributions from CIP adsorption (−15 to −20 mPt) and regeneration (25 to −30 mPt) significantly reduce the net burden, offsetting roughly ~30–40% of the total impact. On the other hand, for the ecosystem quality category impact, the total impact is around ~10–12 mPt, again dominated by Stage 2 (6–7 mPt; 60–65%) and Stage 4 (3–4 mPt; 30–35%). The adsorption and regeneration stages provide additional credits (3 to −5 mPt combined), resulting in a noticeable reduction in ecosystem-related impacts. Meanwhile, considering the impact on resource scarcity category, the total impact is comparatively low at approximately 3–5 mPt, but still primarily governed by Stage 2 (2–3 mPt; 70–75%) and Stage 4 (1–1.5 mPt; 20–25%). Regeneration contributes the largest environmental credit in this category (1.5 to −2 mPt), offsetting nearly 30–40% of total resource-related impacts, emphasizing the importance of material reuse. The relative contribution analysis (Figure 12b) further confirms that Stage 2 consistently dominates across all endpoint categories, while Stage 4 serves as the secondary contributor, and Stages 5–6 provide consistent negative contributions, improving overall environmental performance.

#### 2.3.3. Hotspot Identification and Mitigation Strategies

**Figure 13:**
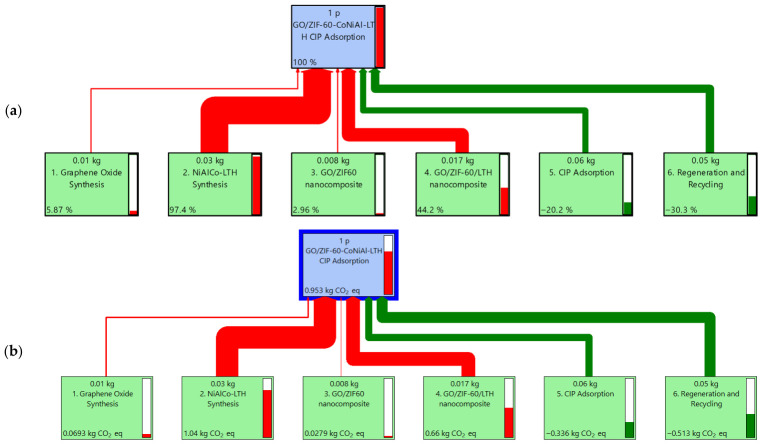
Sankey diagrams illustrating impact contributions of the GO/ZIF-60/CoNiAl-LTH adsorption individual stages (**a**) overall % environmental contributions based on normalized single-score results and (**b**) contributions to global warming potential in kg CO_2_ eq. Impact proportional to arrow thickness; Red connector = net negative impact; Green connector = net positive impact.

The integrated midpoint, endpoint, and Sankey analyses provide a coherent and robust identification of environmental hotspots within the GO/ZIF-60/LTH system, which is strongly supported by findings from previous LCA studies on advanced adsorbents. As shown in the Sankey diagram in Figure 13a,b, NiAlCo-LTH synthesis dominates the environmental profile, contributing approximately 1.04 kg CO_2_ eq (up to 97.4% of total positive burden) and accounting for the highest endpoint damages (90–95 mPt in human health in Figure 12a. This dominance is primarily driven by intensive metal precursor consumption (Ni, Co, Al salts) and associated chemical processing. Similar trends have been consistently reported in the literature, where chemical synthesis and precursor manufacturing are identified as the primary environmental hotspots. Recently, Wang et al. [58] reported that chemical activation (HCl/NaOH) dominated eutrophication and ozone depletion impacts in algal biochar systems, while Gonzalez et al. [59] showed that >60% of impacts in Cd(II) nanoadsorbents originated from precursor synthesis. Likewise, several studies such early-stage nanoadsorbent LCA undertaken by Lui et al. [60], further supported by, chitosan modified Ni-Fe-LDH presented by Bisaria et al. [16] and LDH-MgFe comparative studies da by Silva et al. [61] confirmed that chemical inputs and electricity consumption are the principal drivers of global warming, toxicity, and resource depletion impacts. Furthermore, Garcia Gonzalez et al. [59] identified nanomaterial synthesis as the dominant hotspot due to high electricity demand, reinforcing the consistency of the present findings for wide range of different types of adsorbents.

The second most significant contributor identified previously as the GO/ZIF-60/LTH composite formation, which contributes approximately 0.66 kg CO_2_ eq (44.2%) and 35–40 mPt in human health, mainly due to energy-intensive drying (freeze/thermal) and composite integration processes. This observation is in strong agreement with previous studies where thermal treatment and post-synthesis processing were identified as key environmental hotspots. In this regards, Wang et al. [58] observed that pyrolysis and drying dominated impacts in biochar systems, while other studies showed that post-synthesis processing significantly increased human toxicity and ecosystem damage in alginate-based nanocomposites. Similarly, the LCA of agricultural residue-derived adsorbents Nandikes et al. [59] demonstrated that although biomass feedstocks may reduce some impacts, chemical activation and energy-intensive post-processing remain the dominant contributors [17]. The findings are further supported by Chiew et al. [60], where chemical usage (e.g., HCl) accounted for 79–98% of environmental impacts, and by LDH synthesis studies [57,60], which emphasized the critical role of electricity and chemical consumption in driving environmental burdens. In contrast, upstream stages such as GO synthesis (5.87%, 0.069 kg CO_2_ eq) and GO/ZIF-60 formation (2.96%, 0.028 kg CO_2_ eq) contribute relatively minor impacts, confirming that support material preparation plays a secondary role compared to synthesis and post-processing stages.

A distinguishing and favorable feature of utilizing GO/ZIF-60/LTH for CIP is the substantial environmental credits associated with the application stages, as visualized by the negative flows in the Sankey diagram. Specifically, CIP adsorption and regeneration/recycling offset approximately −20.2% (0.336 kg CO_2_ eq) and −30.3% (~−0.513 kg CO_2_ eq), respectively. Notably, regeneration alone offsets nearly 50% of the emissions from composite synthesis, while the combined effect of Stages 5 and 6 compensates for a significant portion of upstream synthesis burdens. This behavior is consistent with findings from Chiew et al. [60], where regeneration reduced impacts by more than 70%, and highlights the critical importance of adsorbent efficiency and reusability in improving overall environmental performance. Such functional offsets are often underrepresented in conventional LCA studies focused primarily on material production, and their inclusion provides a more realistic and decision-relevant assessment of sustainability.

Based on these insights, several targeted mitigation strategies are proposed. First, reducing the environmental intensity of NiAlCo-LTH synthesis is essential and can be achieved by optimizing metal precursor usage, improving reaction efficiency, and exploring lower-impact alternatives (e.g., Fe-, Mg-, or Zn-based systems), as suggested in LDH-related studies. Additionally, chemical recovery and recycling can significantly reduce toxicity and resource-related disposal impacts. Second, the environmental burden associated with Stage 4 can be mitigated through energy optimization strategies, such as replacing freeze-drying with lower-energy drying methods, optimizing operating conditions, and integrating renewable or waste heat sources, thereby reducing global warming impacts. Third, enhancing adsorption capacity, regeneration efficiency, and material durability is critical to maximizing environmental credits and improving lifecycle performance. Finally, process intensification and integration—including reducing washing steps, minimizing solvent usage, and adopting one-pot synthesis approaches—can further decrease cumulative environmental burdens.

Generally, the GO/ZIF-60/LTH system demonstrates a clear environmental trade-off between high-impact synthesis stages (Stages 2 and 4) and beneficial adsorption and regeneration stages (Stages 5 and 6). This pattern is consistent with findings across several types of adsorbents and underscores that achieving sustainable performance requires a combined optimization strategy: reducing chemical and energy intensity during synthesis while maximizing adsorption efficiency and regeneration capacity. Such an approach enables substantial mitigation of lifecycle impacts and supports the development of more sustainable and scalable adsorbent technologies for pharmaceutical-contaminated water treatment. Interestingly, the LTH phase that contributed most significantly to the environmental impacts was also responsible for providing additional active sites, electrostatic interactions, and coordination mechanisms that enhanced ciprofloxacin adsorption. This highlights a trade-off between adsorption performance and environmental burden, emphasizing the importance of optimizing synthesis conditions to maximize functionality while minimizing environmental impacts.

Although in this study, the GO/ZIF-60/LTH composite demonstrated excellent adsorption performance, yet further evaluation of production cost, process scalability, and long-term environmental safety are needed for understanding its full application potentails. In particular, potential leaching of Co, Ni, Zn, and Al species from nanocomposites under different operating conditions should be assessed to ensure environmental compatibility. In addition, techno-economic and pilot-scale studies are needed to evaluate the feasibility of large-scale deployment. In this regard, the LCA conducted in this study provides an initial sustainability perspective and identifies opportunities for reducing environmental burdens during material synthesis.

### 2.4. Mechanisms of Ciprofloxacin Adsorption onto GO/ZIF-60/LTH

**Figure 14:**
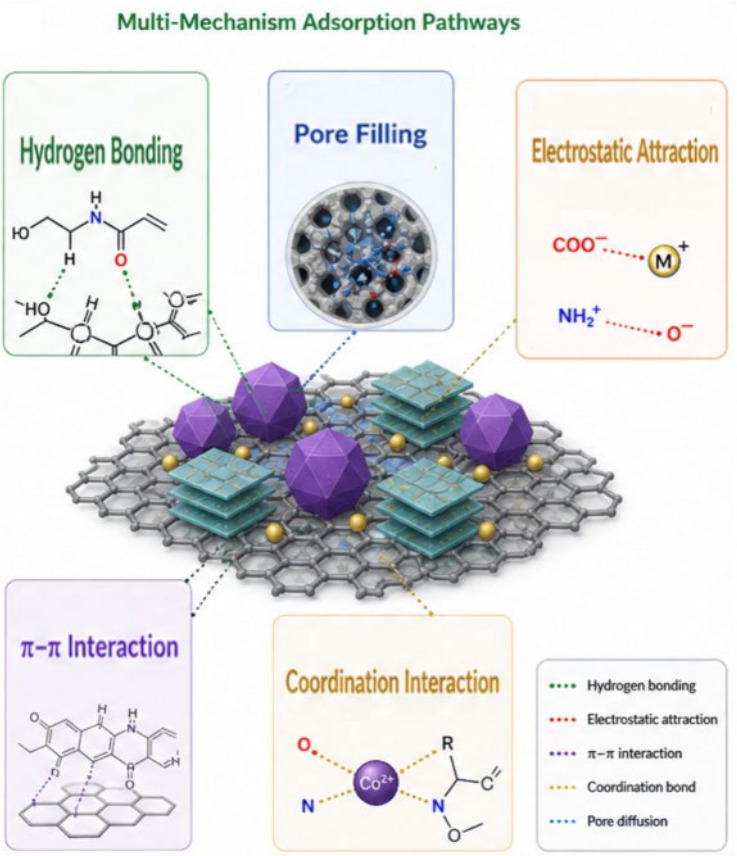
Different Mechanisms Involved for Ciprofloxacin adsorption onto GO/ZIF-60/LTH.

The high adsorption performance of the GO/ZIF-60/LTH nanocomposite can be attributed to the synergistic contribution of multiple adsorption mechanisms (Figure 14), consistent with the characterization and adsorption results presented earlier [14]. FTIR analysis confirmed the presence of hydroxyl, carboxyl, imidazole, and metal-associated functional groups that provide active sites for hydrogen bonding and electrostatic interactions with CIP molecules. The optimum adsorption at pH 6 indicates that electrostatic attraction plays an important role when CIP predominantly exists in its zwitterionic form. Other studies have seen similar results when using materials like graphene and layered hydroxides at pH levels close to neutral [48]. In addition, the graphitic structure of GO promotes π–π stacking with the aromatic rings of CIP, while the high surface area and hierarchical porosity of the composite facilitate pore filling and molecular diffusion. Previous studies reported that CIP adsorption onto graphene-based materials is mainly governed by hydrogen bonding, electrostatic attraction, and π–π interactions [62].

The presence of Co, Ni, Zn, and Al metal centers further suggests possible coordination interactions with oxygen- and nitrogen-containing functional groups of ciprofloxacin. In order words, the CoNiAl-LTH part of the composite has areas rich in hydroxyl groups, which can create strong electrostatic attractions and hydrogen bonds with the molecules being absorbed [63]. These findings are supported by the Freundlich isotherm model (R^2^ = 0.98), which indicates heterogeneous multilayer adsorption, and by the pseudo-first-order kinetic model, suggesting that adsorption is primarily controlled by surface interactions. Critically, the results demonstrate that the enhanced adsorption performance is not governed by a single dominant mechanism but rather by the cooperative action of several complementary interactions. This multifunctional adsorption behavior explains why the GO/ZIF-60/LTH composite outperformed the individual components and achieved a high adsorption capacity (291 mg g^−1^) and removal efficiency (91.6%). The coexistence of multiple binding mechanisms also reduces dependence on a single surface property, thereby improving adsorption robustness under varying water chemistry conditions.

From a practical perspective, the composite combines high removal efficiency with good regeneration capability, retaining approximately 75% of its initial performance after three adsorption–desorption cycles. The hierarchical porous structure facilitates rapid pollutant transport, while the diverse active sites enable effective removal of complex pharmaceutical contaminants. Furthermore, the LCA demonstrated that despite the environmental burdens associated with synthesis, the adsorption and regeneration stages provide measurable environmental credits, supporting the potential application of GO/ZIF-60/LTH as a sustainable adsorbent for pharmaceutical wastewater treatment and future scale-up applications.

## 3. Materials and Methods

### 3.1. Chemicals and Materials

All chemicals used were of analytical grade and utilized without further purification. Zinc nitrate hexahydrate (Zn(NO_3_)_2_·6H_2_O), imidazole, 2-methylimidazole, graphite powder, potassium permanganate (KMnO_4_), sodium nitrate (NaNO_3_), hydrogen peroxide (H_2_O_2_), and sulfuric acid (H_2_SO_4_) were procured from Sigma-Aldrich (Burlington, VT, USA) Nickel(II) chloride hexahydrate (NiCl_2_·6H_2_O), aluminum chloride nonahydrate (AlCl_3_·9H_2_O), cobalt(II) chloride hexahydrate (CoCl_2_·6H_2_O), and urea were used in the synthesis of NiAlCo-LTH. CIP was obtained in pure analytical form from Avalone Pharma (Riyadh, Saudi Arabia). Deionized water was used throughout all experiments.

### 3.2. Synthesis of ZIF-60, GO and NiAlCo-LTH

#### 3.2.1. Synthesis of ZIF-60

ZIF-60 was synthesized using a solvothermal approach [64]. Initially, 2.68 g of zinc nitrate hexahydrate [Zn(NO_3_)_2_·6H_2_O] was dissolved in 60 mL of N,N-dimethylformamide (DMF) under continuous magnetic stirring. In a separate beaker, 1.84 g of imidazole and 0.74 g of 2-methylimidazole were dissolved in DMF through stirring. The two prepared solutions received one hour of magnetic stirrer treatment at room temperature before their combination. The beaker received aluminum foil coverage to stop environmental moisture from entering during this process. The mixture was placed inside a Teflon-lined stainless steel autoclave for solvothermal Zhengzhou Keda Machinery (Zhengzhou, China) processing at 85 °C for 48 h. Upon completion, the resulting ZIF-60 product was collected and washed several times with DMF using centrifugation benchtop centrifuge Thermo-Fisher brand (Waltham, MA, USA) operated at 5000 rpm, followed by additional washes with deionized water to remove residual impurities. The washed product was then dried in an oven at 50 °C until complete solvent evaporation. Finally, the dry ZIF-60 powder received storage in sealed vials to maintain its structural stability before researchers could utilize it.

#### 3.2.2. Synthesis of Graphene Oxide (GO)

Graphene oxide was prepared using a modified Hummers’ method [65]. The graphite powder underwent oxidation through a process that combined KMnO_4_ with NaNO_3_ and H_2_SO_4_ under specific low-temperature conditions, followed by neutralization and filtration and washing steps. The obtained GO was dried and stored in a desiccator for storage.

#### 3.2.3. Synthesis of NiAlCo-Layered Triple Hydroxide (LTH)

NiAlCo-LTH was synthesized using a traditional hydrothermal approach [29]. Nickel(II) chloride hexahydrate (NiCl_2_·6H_2_O), aluminum chloride nonahydrate (AlCl_3_·9H_2_O), and cobalt(II) chloride hexahydrate (CoCl_2_·6H_2_O) were mixed in a molar ratio of 2:1:2, respectively. The mixture contained 80 mmol of CoCl_2_·6H_2_O, 80 mmol of NiCl_2_·6H_2_O, and 40 mmol of AlCl_3_·9H_2_O dissolved in 30 mL of deionized water under continuous magnetic stirring. Then, the addition of 50 mL of 3 M NaOH solution to the precursor mixture under room temperature stirring conditions allowed pH adjustment to 12 while the solution stirring continued. The resulting suspension was transferred into a 100 mL Teflon-lined stainless steel autoclave for 24 h at 180 °C. After naturally cooling to room temperature, the resulting precipitate was collected by filtration, followed by washing with deionized water and ethanol before drying at 80 °C for an overnight period.

### 3.3. Preparation of GO/ZIF60 and GO/ZIF-60/LTH Composites

#### 3.3.1. Synthesis of GO/ZIF60 Nanocomposite

The GO/ZIF60 nanocomposite was synthesized by first dispersing 0.2 g of graphene oxide (GO), prepared via the Hummers’ method, into 30 mL of ethanol. The suspension was magnetically stirred for 30 min at room temperature to achieve a uniform distribution of GO. The pH of the mixture was then adjusted to the range of 10–11. Subsequently, the same amount of (ZIF60) was introduced to the GO suspension. The resulting mixture underwent ultrasonication for 1 h at 50% amplitude to enhance GO-ZIF60 component bonding [66]. After sonication, the resulting GO/ZIF60 composite was recovered by centrifugation (10,000 rpm) and washed repeatedly with deionized (DI) water to remove all remaining contaminants. The purified product was dried in an oven at 50 °C until complete solvent removal. The dried GO/ZIF60 powder was stored in vials for further use. The GO/LTH composite was synthesized in the same way.

#### 3.3.2. Synthesis of GO/ZIF-60/LTH Nanocomposite

The GO/ZIF 60/LTH nanocomposite was synthesized through a modified solvothermal process that utilized GO/LTH as the starting material. Initially, 2.68 g of zinc nitrate hexahydrate [Zn(NO_3_)_2_·6H_2_O] and 0.5 g of the previously prepared GO/LTH composite were dispersed in 60 mL of N,N-dimethylformamide (DMF), followed by ultrasonication for 1 h at 50% amplitude to ensure thorough mixing and interaction. In another solution, 1.84 g of imidazole and 0.74 g of 2-methylimidazole were mixed with DMF and stirred constantly until they were completely dissolved. The two solutions were then combined and stirred magnetically at room temperature for 1 h. To prevent moisture absorption, the beaker was covered with aluminum foil during this process. The homogeneous mixture received solvothermal treatment at 85 °C for 48 h inside a Teflon-lined stainless-steel autoclave. After cooling to room temperature, the synthesized GO/ZIF 60/LTH composite was collected by centrifugation (10,000 rpm) and washed several times with DMF, followed by repeated rinsing with deionized water to remove any unreacted precursors or residual solvents [26]. The dried product underwent oven heating at 50 °C until it became completely dry before storage in sealed containers for future use.

### 3.4. Characterization Techniques

Several advanced analytical techniques were used to analyze the morphology, crystallinity and functional groups of the new synthesized MOF-based adsorbents. The combination of Scanning Electron Microscopy (SEM) with Energy Dispersive Spectrometry (EDS) employing a JEOL instrument (JEOL, Tokyo, Japan) to evaluate their surface characteristics and elemental compositions. The XRD analysis was undertaken between 5° and 70° 2θ to establish their crystalline structure and phase composition for confirming their structural stability. FTIR spectroscopy was performed using a Bruker TENSOR27 FTIR spectrometer (Bruker, Ettlingen, Germany) in the spectral range of 4000–500 cm^−1^ to identify the surface chemical functional groups developed during the synthesis process. The standard multipoint Brunauer–Emmett–Teller (BET) surface area analysis was performed using a nitrogen adsorption–desorption analyzer to measure pore diameter and volume and specific surface area through nitrogen adsorption desorption isotherms. The analysis revealed detailed information about the porous structure of adsorbents, which helps researchers optimize their adsorption capabilities.

### 3.5. Experimental Metholodogies for CIP Uptake

#### 3.5.1. Experimental Design, RSM Modeling, and Optimization

**Figure 15:**
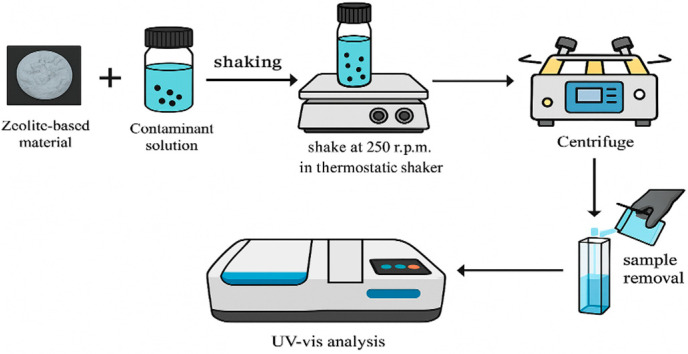
Schematic illustration of the adsorption experiment workflow and UV–Vis analysis.

Optimization of CIP removal from water was performed with the aid of Design-Expert^®^ Version 8 software based on Response Surface Methodology (RSM) employing a Box–Behnken experimental Design (BBD). Three independent variables, namely contact time (60–300 min), initial CIP concentration (10–50 mg L^−1^), and temperature (25–45 °C), were evaluated at three levels, while the solution pH was maintained constant at pH 6, corresponding to the optimum value identified in the preliminary adsorption experiments. The experiments operational factors and their levels are presented in Table 8. A total of seventeen (17) experimental runs were required by implementing the BBD experimental matrix. Batch adsorption experiments were conducted in a temperature-controlled incubator shaker (Biobase, Fremont, CA, USA) at the desired operating conditions and an agitation speed carried out as illustrated in Figure 15. In each run, a 10 mg of GO/ZIF-60/LTH adsorbent was added to the CIP solution and agitated at 250 rpm using a thermostatic shaker to ensure adequate contact between the adsorbent and adsorbate molecules. After the specified contact time, the suspensions were centrifuged at 10,000 rpm to separate the adsorbent from the solution, and the residual CIP concentration was determined using UV–Vis spectrophotometry and corresponding removal efficiencies were then calculated (as detailed in Section 3.5.2) and used as the response variable RSM model development To achieve that, the experimental data were fitted to a second-order polynomial model, and analysis of variance (ANOVA) was performed to evaluate model significance and the effects of individual and interaction terms. Model adequacy and predictive capability were assessed using the coefficient of determination (*R*^2^), adjusted *R*^2^, predicted *R*^2^, lack-of-fit test, *F*-value, *p*-value, and adequate precision. The validated model was subsequently employed to generate three-dimensional (3D) response surface plots and two-dimensional (2D) contour plots for visualization of interaction effects and optimization of adsorption conditions. This approach enabled identification of the optimum operating conditions for CIP removal while providing insight into the interactive influence of process variables on adsorption performance via statistical ANOVA.

#### 3.5.2. Measurement of Ciprofloxacin Uptake

Standard CIP solutions with initial concentrations of 10, 30, and 50 mg L^−1^ were prepared by diluting a 1000 mg L^−1^ stock solution with deionized water. Following adsorption (Section 3.5.1), the adsorbent particles were separated by centrifugation at 10,000 rpm, and the residual CIP concentration in the supernatant was determined using a UV–Vis spectrophotometer HACH DR 6000 (Hach Company, Loveland, CO, USA) at the maximum absorption wavelength (λmax = 270 nm) [67,68]. A calibration curve was established using standard CIP solutions of known concentrations, and the residual concentrations were quantified from the corresponding absorbance values. The adsorption performance was evaluated in terms of removal efficiency (%) and adsorption capacity, calculated using Equations (2) and (3), respectively:

(2) $$\mathrm{Removal}\;(\%)\;=\;\frac{C_{0}\;-\;C_{e}}{C_{0}}\;\times \;100$$

where Co and Ce are initial and equilibrium concentrations (mg L^−1^), respectively.

The adsorption capacity (qt) of the adsorbents was calculated by Equation (3).

(3) $$qt=\frac{(C_{0}\;-\;C_{e})\times V}{W}$$

where V is the volume (L) of the solution and *W* is the mass (g) of the adsorbent [52].

#### 3.5.3. Adsorption Kinetics Study

Adsorption kinetic experiments were conducted to investigate the rate and mechanism of CIP uptake onto the GO/ZIF-60/LTH nanocomposite. About 10 mg of adsorbent was added to 40 mL of CIP solution in conical flasks and agitated under the predetermined experimental conditions. At specified time intervals, 4 mL aliquots were withdrawn, filtered, and analyzed for residual CIP concentration using the procedure described previously. The amount of CIP adsorbed at time t (qt, mg g^−1^) was calculated and fitted to the pseudo-first-order, pseudo-second-order, intra-particle diffusion, and Elovich kinetic models in Equations (4)–(7), respectively, to elucidate the adsorption mechanism and rate-controlling steps.


(4) $$q_{t}=q_{e}(1-e^{-k_{1}t})$$

(5) $$q_{t}=\frac{{q_{e}}^{2}\;K_{2}\;t}{1+q_{e}\;K_{2}\;t}$$

(6) $$q_{t}=K_{\mathrm{id}}\cdot t_{0.5}+C$$

(7) $$qt=\frac{1}{\beta }ln(\alpha \beta )+\frac{1}{\beta }ln(t)$$

The adsorption rate constants, for the pseudo-first order, pseudo-second order models, Intra-Particle Diffusion, and Elovich are denoted as K_1_ (min^−1^), K_2_ (g mg^−1^ min^−1^) and k_id_ (mg/g·min^0.5^, respectively, t (min) represents the adsorption time, α is the initial adsorption rate (mg/g·min), and β is the desorption constant (g/mg).

#### 3.5.4. Adsorption Isotherm Study

Adsorption isotherm experiments were conducted to evaluate the equilibrium behavior of CIP adsorption onto the GO/ZIF-60/LTH nanocomposite. Batch adsorption tests were performed using 40 mL of CIP solutions with varying initial concentrations while maintaining constant adsorbent dosage, contact time, pH, and temperature. Following equilibration, the residual CIP concentration was determined, and the equilibrium adsorption capacity (q_e_) was calculated. The experimental data were analyzed using the Langmuir, Freundlich, and Temkin isotherm models in Equations (8)–(10), respectively, to elucidate the adsorption mechanism and surface characteristics [1].


(8) $$\mathrm{qe}=\frac{KLqmCe}{1+KLCe}$$

(9) $$\mathrm{qe}\;=\;{{\mathrm{KFC}}_{e}}^{1/n}$$

(10) $$\mathrm{qe}\;=\;B\;\ln(K_{t}C_{e})$$

The Langmuir and Freundlich adsorption constants are denoted as K_L_ (L mg^−1^), K_F_ (mg g^−1^ (L mg^−1^) K, KT (L mg^−1^), respectively. The maximum adsorption amount is represented by qm (mg g^−1^), where the intensity of adsorption is expressed by the value 1/n.

#### 3.5.5. Adsorption Thermodynamic Study

Adsorption thermodynamic experiments were conducted to evaluate the effect of temperature on the adsorption behavior of CIP onto the GO/ZIF-60/LTH nanocomposite. Briefly, 10 mg of adsorbent was added to 40 mL of CIP solution in sealed Erlenmeyer flasks. The mixtures were agitated in a temperature-controlled orbital shaker at 298, 308, and 318 K until equilibrium was reached. The equilibrium adsorption capacity (qe) and equilibrium concentration (Ce) were subsequently used to determine the thermodynamic parameter using Equations (11)–(13).

(11) $$K_{d}=\frac{q_{e}}{C_{e}}$$

(12) $$\Delta G\;=\;-RT\;\ln\;K_{d}$$

(13) $$\ln\;K_{d}=\frac{\Delta S}{R}-\frac{\Delta H}{RT}$$

where K_d_ is the distribution coefficient, qe (mg g^−1^) is the equilibrium adsorption capacity, and Ce (mg L^−1^) is the equilibrium concentration of CIP in solution. In Equation (12), R is the universal gas constant (8.314 J mol^−1^ K^−1^) and T is the absolute temperature (K). The thermodynamic parameters ΔG° (kJ mol^−1^), ΔH° (kJ mol^−1^), and ΔS° (J mol^−1^ K^−1^) represent the standard Gibbs free energy, enthalpy, and entropy changes in the adsorption process, respectively. The values of ΔH° and ΔS° were obtained from the slope and intercept of the linear plot of lnK_d_ versus 1/T.

### 3.6. Recyclability of Adsorbent

The reusability of the GO/ZIF-60/LTH adsorbent was evaluated through consecutive adsorption–desorption cycles. After each adsorption experiment, the adsorbent was separated, washed several times with distilled water and ethanol to remove residual CIP molecules, and then dried at 60 °C before reuse in the next cycle under the same experimental conditions. The adsorption efficiency was calculated after each cycle to evaluate the stability and regeneration performance of the adsorb. The adsorption experiment was repeated 4 times at room temperature using the dried sample, and the recyclability was evaluated based on the amount of cyclic adsorption.

### 3.7. Life Cycle Assessment of GO-ZIF60-LTH Synthesis and CIP Adsorptive Application

The life cycle assessment (LCA) of the GO/ZIF-60/LTH nanocomposite was conducted in accordance with ISO 14044 using SimaPro 9.1 [69], the Ecoinvent v3.8 database, and the ReCiPe 2016 v1.13 method [18]. A cradle-to-gate life cycle inventory (LCI) was developed covering GO preparation, ZIF-60 synthesis, NiAlCo-LTH formation, composite integration, washing, and drying processes. The LCA would enable quantification of material and energy flows, identification of environmental hotspots, and evaluation of sustainability trade-offs associated with the synthesis and application of the adsorbent for CIP removal from water.

#### 3.7.1. Goal and Scope Definition

The LCA was undertaken to quantify the cradle-to-gate environmental impacts associated with the synthesis and application of the GO/ZIF-60/LTH nanocomposite for CIP adsorption [18]. The assessment aims to identify environmental hotspots across the integrated material synthesis–application pathway and to evaluate the sustainability implications of developing a multi-component hybrid adsorbent system [16]. The functional unit (FU) is defined as the removal of 1 g of CIP from aqueous solution, accounting for adsorption performance and regeneration efficiency over multiple reuse cycles. All material and energy inputs are normalized to this functional unit. The system boundary -defined as cradle-to-gate with application within the process sequence illustrated in Figure 12- includes the following stages: (i) GO synthesis, (ii) NiAlCo-LTH nanocomposite synthesis, (iii) GO/ZIF-60 synthesis, (iv) GO/ZIF-60/LTH ternary nanocomposite synthesis, (v) adsorption of CIP using GO/ZIF-60/LTH, and (vi) four (4) regeneration and reuse cycles of the adsorbent. These stages collectively represent the full material development and application pathway, capturing all relevant inputs such as chemical precursors, solvents, water usage, and electricity consumption. The regeneration stage is explicitly included to reflect the reuse potential of the adsorbent and its influence on environmental performance per functional unit. Excluded from the system boundary are capital equipment, laboratory infrastructure, transportation, infrastructure construction, and end-of-life disposal of the adsorbent, as these are considered negligible at the laboratory scale.

#### 3.7.2. Life Cycle Inventory (LCI) and Impact Assessment

**Figure 16:**
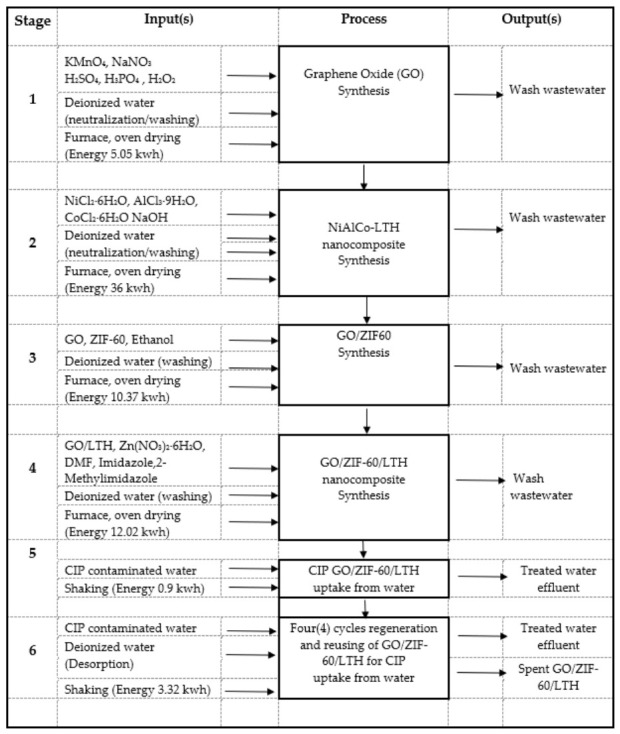
Life Cycle inventory for GO/ZIF-60/LTH synthesis routes and application for CIP adsorption, regeneration and reuse.

The LCI was developed based on experimentally measured laboratory-scale material inputs and estimated electricity consumption for each unit operation involved in the synthesis and adsoprtive application of the GO/ZIF-60/LTH nanocomposite for CIP uptake from water. The inventory includes mass and energy flows in and out associated with GO synthesis, ZIF-60 formation, NiAlCo-LTH preparation, and subsequent composite integration, as well as adsorption and regeneration processes as illustrated in Figure 16. Accordingly, the key inputs comprised of the chemical precursors (metal salts, organic linkers), solvents, deionized water (as detailed in the methodology sections), and electricity consumption associated with ultrasonication, mixing, heating, centrifugation, washing, and drying operations across all stages involved in the GO/ZIF-60/LTH nanocomposite synthesis and application. Electricity consumption for each process stage was calculated based on equipment rated power, operating time, and duty cycle according to Equation (14):

(14) E=P(W)×Duty×t(h)/1000

where E is the electricity consumption (kWh), P is the equipment power (W), and t is the operating time (h). All energy inputs were modeled using the relevant electricity mix available in the Ecoinvent database. Water use was modeled as deionized water consumption without wastewater treatment credits.

The LCI data were modeled in SimaPro 9.1 using the Ecoinvent v3.8 database and the ReCiPe 2016 method at both midpoint and endpoint levels [18]. Midpoint indicators provided detailed assessment of specific environmental impacts, while endpoint results aggregated these impacts into the damage categories of human health, ecosystem quality, and resource scarcity. Normalization and weighting were applied using the ReCiPe hierarchist perspective to generate single-score results (mPt), facilitating hotspot identification and comparison of life-cycle stages. All inventory flows were normalized to the functional unit of removing 1 g of CIP, incorporating adsorption efficiency and regeneration performance to provide a realistic assessment of environmental sustainability [18].

## 4. Conclusions

In this study, novel ZIF-60-based adsorbents and their binary and ternary composites with graphene oxide (GO) and layered triple hydroxide (LTH) were synthesized, characterized, optimized using Response Surface Methodology (RSM), evaluated for CIP adsorption, and assessed from both performance and environmental sustainability perspectives through life cycle assessment (LCA). Among the prepared materials, GO/ZIF-60/LTH exhibited the best performance, achieving a maximum adsorption capacity of 291 mg g^−1^ and a removal efficiency of 91.6%, outperforming its individual and binary counterparts. Adsorption followed pseudo-first-order kinetics and the Freundlich isotherm model, indicating heterogeneous multilayer adsorption, while thermodynamic analysis confirmed a spontaneous and exothermic process. The enhanced performance was attributed to the synergistic effects of hydrogen bonding, electrostatic attraction, π–π interactions, pore filling, and metal–ligand coordination. The composite also demonstrated good reusability, retaining approximately 75% of its initial efficiency after three regeneration cycles. The environmental evaluation revealed that the synthesis of GO/ZIF-60/LTH is associated with a global warming impact of approximately 0.953 kg CO_2_ eq per functional unit (1 g CIP removed). The analysis identified NiAlCo-LTH synthesis as the dominant environmental hotspot (1.04 kg CO_2_ eq; ~97.4% contribution), followed by composite formation (0.66 kg CO_2_ eq; 44.2% contribution), primarily driven by metal precursor usage and energy-intensive processing steps such as drying and integration. In contrast, adsorption and regeneration stages provide significant environmental credits (−0.336 and −0.513 kg CO_2_ eq), offsetting a substantial portion of upstream impacts and demonstrating the importance of adsorbent reusability in improving overall environmental performance. The combined experimental and life cycle assessment results demonstrate that GO/ZIF-60/LTH offers a favorable balance between adsorption performance and environmental sustainability. Although NiAlCo-LTH synthesis represents the primary environmental hotspot, the high adsorption capacity, efficient CIP removal, and regeneration capability provide environmental benefits that partially compensate for these impacts.

## Figures and Tables

**Table 1:** BET results of synthesis materials.

Sample ID	Surface Area (m^2^ g^−1^)	Pore Volume (cm^3^ g^−1^)	Pore Diameter (nm)
**GO**	567.4	3.636	1.28
**LTH**	363.5	2.040	1.12
**ZIF-60**	4213.4	4.059	1.92
**GO/ZIF-60**	646.6	1.788	5.50
**GO/ZIF-60/LTH**	568.9	2.810	9.87

**Table 2:** Results of Box–Behnken (BDD) design matrix for GO/ZIF60/CoNiAl-LTH Ciprofloxacin removal from water experiments.

Run	A:Contact Time Min	B:Concentration Ppm	C:Temperature °C	Removal Efficiency %
1	180	50	35	80.161
2	300	10	45	85.824
3	60	10	25	82.848
4	180	30	35	86.976
5	60	50	45	74.112
6	180	10	35	91.68
7	300	10	25	83.616
8	180	30	35	86.88
9	300	50	25	72.768
10	60	30	35	81.312
11	60	30	25	77.184
12	300	30	35	81.792
13	60	50	25	73.44
14	300	30	45	76.416
15	180	30	35	87.168
16	60	10	45	84.48
17	300	50	45	75.456

**Table 3:** Adsorption kinetics parameters of GO/ZIF-60/LTH.

Pseudo-First-Order			Pseudo-Second-Order		
*k*_1_ (min^−1^)	*q_e__cal*	*R* ^2^	*k*_2_ (mg g^−1^ min^−1^)	*q_e__cal*	*R* ^2^
0.00892	34.9	0.97212	2.78	39.42	0.9509
**Intra-particle diffusion**			**Elovich model**		
*K_p_* (mg g^−1^ min^−0.5^)	*C*	*R* ^2^	*K_f_* (min^−1^)	*A*	*R* ^2^
0.968	7.489	0.677	0.135	1.054	0.898

**Table 4:** Adsorption isotherm parameters of GO/ZIF-60/LTH.

Langmuir Adsorption Isotherm q_e_ = (q_max_ K_l_ C_e_)/(1 + K_l_ C_e_)			Freundlich Adsorption Isotherm q_e_ = K_f_ C_e_^(1/n)^			Temkin Adsorption Isotherm q_e_ = B ln(K_T_C_e_)		
q_max_	*K*_l_	*R* ^2^	N	*K*_f_	*R* ^2^	B	K_T_	*R* ^2^
291	0.22	0.89	1.46	10.11	0.98	71.1	0.263	0.9315

**Table 5:** Thermodynamics parameters for ciprofloxacin uptake by GO/ZIF-60/LTH.

Pollutant	T (K)	ΔG (kJ mol^−1^)	ΔH (kJ mol^−1^)	ΔS (J mol^−1^ k^−1^)
Ciprofloxacin	298	−4.80669	−68.3669	−214.16
	308	−1.84825		
	318	−0.55956		

**Table 6:** Comparative assessment of different adsorbents for removal of CIP.

Adsorbent	Maximum Adsorption Capacity (mg/g)	Maximum Removal (%)	Reference
Fe/Ni-MOF	Not specified	94.13	[45]
Zn–Fe LDH/Chia Seed Composite	850	85.2	[46]
C@Silica core/shell (ZIF-8)	1.575	59	[47]
Protein-Modified Nanosilica (ProMNS)	85	90	[48]
GO-ZnAlNi LDH	106.97	80	[49]
Fe_3_O_4_/Graphene/Bentonite	236.8	~95	[50]
ZnCo-ZIF@CS beads	348.9	85.3	[51]
Chitosan/Fe–Cu CNS/CMC–Alginate	485.6	90	[52]
HAP/MIL-101(Fe)/Fe3O4	112.4	93	[53]
Fullerene/MgO	Not specified	84.6	[54]
CuCoFe_2_O_4_@AC	Not specified	95.8	[55]
Fe_3_O_4_/GO/citrus peel biochar	283.4	Not specified	[56]
3D MgAl-LDH/rGO	775.2	Not specified	[57]
CMC/CoNiFe-LDH/ZIF-8 (CLZ-1)	1397.5	Not specified	[15]
GO/ZIF-60/LTH (this work)	291	91.5	This study

**Table 7:** Equivalent Environmental impact assessments equivalent for different major midpoint categories for GO/ZIF-60/LTH synthesis route.

Impact Category	Unit	Total	1. GO Synthesis	2. NiAlCo-LTH Synthesis	3. GO/ZIF60 Synthesis	4. GO/ZIF-60/LTH Synthesis	5. CIP Adsorption	6. Regeneration and Recycling
Global warming	kg CO_2_ eq	0.953	0.069	1.044	0.028	0.660	−0.336	−0.513
Stratospheric ozone depletion	kg CFC11 eq	-	-	-	-	-	-	-
Ionizing radiation	kBq Co-60 eq	0.247	0.001	0.243	0.001	0.016	−0.006	−0.008
Ozone formation, Human health	kg NOx eq	0.005	-	0.003	-	0.001	−0.001	0.001
Fine particulate matter formation	kg PM2.5 eq	0.003	-	0.002	-	0.001	-	-
Ozone formation, Terrestrial ecosystems	kg NOx eq	0.007	-	0.004	-	0.001	−0.001	0.002
Terrestrial acidification	kg SO_2_ eq	0.008	-	0.007	0.001	0.002	−0.001	−0.001
Freshwater eutrophication	kg P eq	-	-	-	-	-	-	-
Marine eutrophication	kg N eq	-	-	-	-	-	-	-
Terrestrial ecotoxicity	kg 1,4-DCB	16.130	0.898	35.469	0.784	7.145	−12.556	−15.610
Freshwater ecotoxicity	kg 1,4-DCB	0.003	-	0.004		0.001	−0.001	−0.001
Marine ecotoxicity	kg 1,4-DCB	43.767	1.816	39.946	0.875	10.547	−4.081	−5.336
Human carcinogenic toxicity	kg 1,4-DCB	0.260	0.032	0.213	0.004	0.033	−0.010	−0.013
Human non-carcinogenic toxicity	kg 1,4-DCB	44.338	1.833	37.451	0.723	8.801	−1.894	−2.575
Land use	m^2^a crop eq	0.187	0.005	0.054	0.126	0.006	−0.002	−0.002
Mineral resource scarcity	kg Cu eq	0.481	0.005	0.476	-	-	-	-
Fossil resource scarcity	kg oil eq	0.188	0.023	0.370	0.019	0.277	−0.200	−0.301
Water consumption	m^3^	0.160	0.002	0.151	0.002	0.007	−0.001	−0.002

**Table 8:** Operational variables and their respective levels employed for the RSM BDD experimental design.

Variable Factors		Fixed Factors
Adsorbent dose (mg)		10 mg
pH		6–7
Contact time(minutes)	60, 180 and 300	
Temperature, oC	25, 30 and 45	
Concentrations, mg/L	10, 30 and 50	

## Data Availability

The original contributions presented in this study are included in the article. Further inquiries can be directed to the corresponding authors.

## References

[1] Gahrouei A.E., Vakili S., Zandifar A., Pourebrahimi S. (2024). From wastewater to clean water: Recent advances on the removal of metronidazole, ciprofloxacin, and sulfamethoxazole antibiotics from water through adsorption and advanced oxidation processes (AOPs). Environ. Res..

[2] Rodriguez-Mozaz S., Vaz-Moreira I., Della Giustina S.V., Llorca M., Barceló D., Schubert S., Berendonk T.U., Michael-Kordatou I., Fatta-Kassinos D., Martinez J.L. (2020). Antibiotic residues in final effluents of European wastewater treatment plants and their impact on the aquatic environment. Environ. Int..

[3] Sharma S., Chauhan A., Ranjan A., Mathkor D.M., Haque S., Ramniwas S., Tuli H.S., Jindal T., Yadav V. (2024). Emerging challenges in antimicrobial resistance: Implications for pathogenic microorganisms, novel antibiotics, and their impact on sustainability. Front. Microbiol..

[4] Khan P., Saha R., Halder G. (2024). Towards sorptive eradication of pharmaceutical micro-pollutant ciprofloxacin from aquatic environment: A comprehensive review. Sci. Total Environ..

[5] Mahmud F., Banhi T.S., Roy H., Dihan M.R., Islam M.S., Cai Y., Asiri A.M., Rahman M.M., Hasan M.M., Shenashen M. (2024). Antibiotic-contaminated wastewater treatment and remediation by electrochemical advanced oxidation processes (EAOPs). Groundw. Sustain. Dev..

[6] Mathur P., Sanyal D., Callahan D.L., Conlan X.A., Pfeffer F.M. (2021). Treatment technologies to mitigate the harmful effects of recalcitrant fluoroquinolone antibiotics on the environ-ment and human health. Environ. Pollut..

[7] Thai V.-A., Thuy N.T., Pandit B., Vo T.-K.-Q., Khedulkar A.P. (2023). Fluoroquinolones: Fate, effects on the environment and selected removal methods. J. Clean. Prod..

[8] Park K.S., Ni Z., Côté A.P., Choi J.Y., Huang R., Uribe-Romo F.J., Chae H.K., O’Keeffe M., Yaghi O.M. (2006). Exceptional chemical and thermal stability of zeolitic imidazolate frameworks. Proc. Natl. Acad. Sci..

[9] Ajala O.A., Akinnawo S.O., Bamisaye A., Adedipe D.T., Adesina M.O., Okon-Akan O.A., Adebusuyi T.A., Ojedokun A.T., Adegoke K.A., Bello O.S. (2023). Adsorptive removal of antibiotic pollutants from wastewater using biomass/biochar-based adsorbents. RSC Adv..

[10] Mo Z., Tai D., Zhang H., Shahab A. (2022). A comprehensive review on the adsorption of heavy metals by zeolite imidazole framework (ZIF-8) based nanocomposite in water. Chem. Eng. J..

[11] Ganiyu S.A., Suleiman M.A., Al-Amrani W.A., Usman A.K., Onaizi S.A. (2023). Adsorptive removal of organic pollutants from contaminated waters using zeolitic imidazolate framework Composites: A comprehensive and Up-to-date review. Sep. Purif. Technol..

[12] Ismail U.M., Onaizi S.A., Vohra M.S. (2023). Aqueous Pb (II) removal using ZIF-60: Adsorption studies, response surface methodology and machine learning predictions. Nanomaterials.

[13] Alkoshab M.Q., Al-Amrani W.A., Drmosh Q.A., Onaizi S.A. (2024). Zeolitic imidazolate framework-8/layered triple hydr (oxide) composite for boosting the adsorptive removal of acid red 1 dye from wastewater. Colloids Surf. A Physicochem. Eng. Asp..

[14] Yasmin S., Azam M.G., Hossain M.S., Akhtar U.S., Kabir M.H. (2024). Efficient removal of ciprofloxacin from aqueous solution using Zn–C battery derived graphene oxide enhanced by hydrogen bonding, electrostatic and π-π interaction. Heliyon.

[15] Li C., Wang F., Xu X., Shi Y., Liang J., Yang R., Liu J., Zhao Z. (2023). A high-capacity malleable cellulose aerogel with layered double hydroxide decorating ZIF-8 for efficient adsorption of ciprofloxacin. Chem. Eng. J..

[16] Bisaria K., Seth C.S., Singh R. (2024). Life cycle assessment of chitosan modified Ni–Fe layered double hydroxide for arsenic (iii) sequestration in aqueous medium: Comparison of the impacts of adsorbent recycling, instrument use and source of energy. Environ. Sci. Adv..

[17] Arfasa G.F., Tilahun Z.A. (2025). Life-Cycle impacts of biochar, MOFs, and biomass adsorbents: A meta-analysis for wastewater and carbon management. Environ. Chall..

[18] Oele M., Dolfing R. (2020). SimaPro. https://simapro.com/wp-content/uploads/2020/10/FullUpdateInstructionsToSimaPro911.pdf.

[19] Nandikes G., Nguyen A.H., Siddiqui S.I., Oh S. (2025). Sustainable water treatment using agricultural residue Adsorbents: Evaluating Efficacy and life cycle impacts. J. Ind. Eng. Chem..

[20] Gong Y., Li D., Fu Q., Pan C. (2015). Influence of graphene microstructures on electrochemical performance for supercapacitors. Prog. Nat. Sci. Mater. Int..

[21] Mu’azu N.D., Jarrah N., Kazeem T.S., Zubair M., Al-Harthi M. (2018). Bentonite-layered double hydroxide composite for enhanced aqueous adsorption of Eriochrome Black T. Appl. Clay Sci..

[22] Beigzadeh B., Bahrami M., Amiri M.J., Mahmoudi M.R. (2020). A new approach in adsorption modeling using random forest regression, Bayesian multiple linear regression, and multiple linear regression: 2, 4-D adsorption by a green adsorbent. Water Sci. Technol..

[23] Xu F., Kou L., Jia J., Hou X., Long Z., Wang S. (2013). Metal–organic frameworks of zeolitic imidazolate framework-7 and zeolitic imidazolate framework-60 for fast mercury and methylmercury speciation analysis. Anal. Chim. Acta.

[24] Alagha O., Manzar M.S., Zubair M., Anil I., Mu’azu N.D., Qureshi A. (2020). Magnetic Mg-Fe/LDH Intercalated Activated Carbon Composites for Nitrate and Phosphate Removal from Wastewater: Insight into Behavior and Mechanisms. Nanomaterials.

[25] Suleiman M.A., Zaini M.A.A., Mu’azu N.D. (2025). Pomegranate peel adsorbents for water pollutants removal: Preparation, characterization and applications. Int. J. Phytoremediation.

[26] Bahadi S.A., Drmosh Q., Onaizi S.A. (2024). Adsorptive removal of organic pollutants from aqueous solutions using novel GO/bentonite/MgFeAl-LTH nanocomposite. Environ. Res..

[27] Younis S.R., Abdelmotallieb M., Ahmed A.S. (2025). Facile synthesis of ZIF-8@ GO composites for enhanced adsorption of cationic and anionic dyes from their aqueous solutions. RSC Adv..

[28] Al-Qadri A.A., Drmosh Q., Onaizi S.A. (2022). Enhancement of bisphenol a removal from wastewater via the covalent functionalization of graphene oxide with short amine molecules. Case Stud. Chem. Environ. Eng..

[29] Samadi-Maybodi A., Ghezel-Sofla H., BiParva P. (2023). Co/Ni/Al-LTH layered triple hydroxides with zeolitic imidazolate frameworks (ZIF-8) as high efficient removal of diazinon from aqueous solution. J. Inorg. Organomet. Polym. Mater..

[30] Jun B.-M., Kim S., Kim Y., Her N., Heo J., Han J., Jang M., Park C.M., Yoon Y. (2019). Comprehensive evaluation on removal of lead by graphene oxide and metal organic framework. Chemosphere.

[31] Ayati A., Shahrak M.N., Tanhaei B., Sillanpää M. (2016). Emerging adsorptive removal of azo dye by metal–organic frameworks. Chemosphere.

[32] Firouzjaei M.D., Afkhami F.A., Esfahani M.R., Turner C.H., Nejati S. (2020). Experimental and molecular dynamics study on dye removal from water by a graphene oxide-copper-metal organic framework nanocomposite. J. Water Process Eng..

[33] Montgomery D.C. (2017). Design and Analysis of Experiments.

[34] Mu’azu N.D., AlAmri A.H., Alhamed I.H., Zubair M., Manzar M.S., Nawaz M. (2025). Repurposing of End-of-Life Dialysate Production Polymeric Membrane for Achieving Sustainable Hemodialysis Process Water Management. Polymers.

[35] Langmuir I. (1918). The adsorption of gases on plane surfaces of glass, mica and platinum. J. Am. Chem. Soc..

[36] Ighalo J.O., Rangabhashiyam S., Adeyanju C.A., Ogunniyi S., Adeniyi A.G., Igwegbe C.A. (2022). Zeolitic imidazolate frameworks (ZIFs) for aqueous phase adsorption–a review. J. Ind. Eng. Chem..

[37] Lyu J., Zhang N., Liu H., Zeng Z., Zhang J., Bai P., Guo X. (2017). Adsorptive removal of boron by zeolitic imidazolate framework: Kinetics, isotherms, thermodynamics, mechanism and recycling. Sep. Purif. Technol..

[38] Yu X., Choi S., Tang D., Medford A.J., Sholl D.S. (2021). Efficient models for predicting temperature-dependent Henry’s constants and adsorption selectivities for diverse collections of molecules in metal–organic frameworks. J. Phys. Chem. C.

[39] Freundlich H.M.F. (1906). Over the adsorption in solution. J. Phys. Chem..

[40] Liu Y., Pang H., Wang X., Yu S., Chen Z., Zhang P., Chen L., Song G., Alharbi N.S., Rabah S.O. (2021). Zeolitic imidazolate framework-based nanomaterials for the capture of heavy metal ions and radionuclides: A review. Chem. Eng. J..

[41] Temkin M. (1940). Kinetics of ammonia synthesis on promoted iron catalysts. Acta Physiochim. URSS.

[42] Ma S., Si Y., Wang F., Su L., Xia C., Yao J., Chen H., Liu X. (2017). Interaction processes of ciprofloxacin with graphene oxide and reduced graphene oxide in the presence of montmorillonite in simulated gastrointestinal fluids. Sci. Rep..

[43] Pal B., Singh S., Bansal M. (2024). Superior adsorptive removal of ciprofloxacin by graphene oxide modified Ni-Al layered double hydroxide composites. J. Alloys Compd..

[44] Choi J.-W., Choi S.-J. (2022). Polyacrylamide functionalized graphene oxide/alginate beads for removing ciprofloxacin antibiotics. Toxics.

[45] Wei F., Wang K., Li W., Ren Q., Qin L., Yu M., Liang Z., Nie M., Wang S. (2023). Preparation of Fe/Ni-MOFs for the adsorption of ciprofloxacin from wastewater. Molecules.

[46] Mahgoub S.M., Rudayni H.A., Allam A.A., Alsalamah S.A., Elrafey A., Abdelazeem R., Kotp A.A., Abdelsatar M.M., Shafi R., Mahmoud R. (2025). Green removal and waste valorization of ciprofloxacin from water using zinc–iron LDH–chia seed biocomposites: Integrated adsorption, computational modeling, and electrochemical conversion. RSC Adv..

[47] Malakootian M., Faraji M., Malakootian M., Nozari M. (2021). Ciprofloxacin removal from aqueous media by adsorption process: A systematic review and meta-analysis. Desalination Water Treat..

[48] Pham T.D., Vu T.N., Nguyen H.L., Le P.H.P., Hoang T.S. (2020). Adsorptive removal of antibiotic ciprofloxacin from aqueous solution using protein-modified nanosilica. Polymers.

[49] Sikri N., Kumar S., Behera B., Mehta J. (2025). Graphene oxide/layered double hydroxide composite as highly efficient and recyclable adsorbent for removal of ciprofloxacin from aqueous phase. Front. Nanotechnol..

[50] Alamier W.M., Imran M., Ali S.K., Almashnowi M.Y., Bakry A.M. (2025). Synthesis of high-performance Fe3O4/Graphene/Bentonite clay nanocomposite for sustainable ciprofloxacin adsorption. Inorg. Chem. Commun..

[51] Luo Q., Liu P., Bi L., Shi L., Zhou J., Fang F., Lv Q., Fu H., Li X., Li J. (2024). Selective and efficient removal of ciprofloxacin from water by bimetallic MOF beads: Mechanism quantitative analysis and dynamic adsorption. Sep. Purif. Technol..

[52] Gopal G., Nirmala M.J., Mukherjee A. (2023). A novel chitosan-coated Fe–Cu CNS loaded with CMC–Alginate composite for adsorptive removal of ciprofloxacin from water. Surf. Interfaces.

[53] Beiranvand M., Farhadi S., Mohammadi-Gholami A. (2022). Adsorptive removal of tetracycline and ciprofloxacin drugs from water by using a magnetic rod-like hydroxyapatite and MIL-101 (Fe) metal–organic framework nanocomposite. RSC Adv..

[54] Bekhit S.M., Zaki S.A., Hassouna M.S.E.-D., Elkady M. (2025). Effectiveness of fullerene/magnesium oxide nanocomposite in removing ciprofloxacin and tetracycline from aqueous solutions. RSC Adv..

[55] Pourshaban-Mazandarani M., Ahmadian M., Nasiri A., Poormohammadi A. (2023). CuCoFe2O4@ AC magnetic nanocomposite as a novel heterogeneous Fenton-like nanocatalyst for Ciprofloxacin degradation from aqueous solutions. Appl. Water Sci..

[56] Zhou Y., Cao S., Xi C., Li X., Zhang L., Wang G., Chen Z. (2019). A novel Fe3O4/graphene oxide/citrus peel-derived bio-char based nanocomposite with enhanced adsorption affinity and sensitivity of ciprofloxacin and sparfloxacin. Bioresour. Technol..

[57] Han X.-W., Guo S., Gao X., Lu C., Wang S. (2024). Three-dimensional MgAl layered double hydroxide decorated reduced graphene oxide nanocomposite: An efficient adsorbent for the removal of methylene blue and ciprofloxacin. Appl. Clay Sci..

[58] Wang S., Lu W., Esakkimuthu S., Chen H., Yang J., Mu M., Gong X. (2023). Life cycle assessment of carbon-based adsorbent preparation from algal biomass. J. Clean. Prod..

[59] Gonzalez M.N.G., Quiroga-Flores R., Börjesson P. (2022). Life cycle assessment of a nanomaterial-based adsorbent developed on lab scale for cadmium removal: Comparison of the impacts of production, use and recycling. Clean. Environ. Syst..

[60] Liu R., Zhao Y., Yang Y., Awe O.W. (2018). Diagnosis and evaluation of an early-stage green bio-sorption reactor by life cycle assessment. J. Clean. Prod..

[61] da Silva A.F., da Silva Duarte J.L., Selvasembian R., Meili L. (2023). Life cycle assessment of LDH-MgFe production for nitrate removal: Impacts of synthesis methods. J. Nanoparticle Res..

[62] Huang X., Tian J., Li Y., Yin X., Wu W. (2020). Preparation of a three-dimensional porous graphene oxide–kaolinite–poly (vinyl alcohol) composite for efficient adsorption and removal of ciprofloxacin. Langmuir.

[63] Peng X., Hu F., Lam F.L., Wang Y., Liu Z., Dai H. (2015). Adsorption behavior and mechanisms of ciprofloxacin from aqueous solution by ordered mesoporous carbon and bamboo-based carbon. J. Colloid Interface Sci..

[64] Banerjee R., Phan A., Wang B., Knobler C., Furukawa H., O’Keeffe M., Yaghi O.M. (2008). High-throughput synthesis of zeolitic imidazolate frameworks and application to CO2 capture. Science.

[65] Shahriary L., Athawale A.A. (2014). Graphene oxide synthesized by using modified hummers approach. Int. J. Renew. Energy Environ. Eng..

[66] Singh S., Basavaraju U., Naik T.S.K., Behera S.K., Khan N.A., Singh J., Singh L., Ramamurthy P.C. (2023). Graphene oxide-based novel MOF nanohybrid for synergic removal of Pb (II) ions from aqueous solutions: Simulation and adsorption studies. Environ. Res..

[67] Mohammed A.A., Al-Musawi T.J., Kareem S.L., Zarrabi M., Al-Ma’abreh A.M. (2020). Simultaneous adsorption of tetracycline, amoxicillin, and ciprofloxacin by pistachio shell powder coated with zinc oxide nanoparticles. Arab. J. Chem..

[68] Meng L., Zhao C., Wang T., Chu H., Wang C.-C. (2023). Efficient ciprofloxacin removal over Z-scheme ZIF-67/V-BiOIO3 heterojunctions: Insight into synergistic effect between adsorption and photocatalysis. Sep. Purif. Technol..

[69] (2006). Environmental Management—Life Cycle Assessment—Requirements and Guidelines.

